# Improving access to medicines for non-communicable diseases in rural India: a mixed methods study protocol using quasi-experimental design

**DOI:** 10.1186/s12913-016-1680-3

**Published:** 2016-08-22

**Authors:** N. S. Prashanth, Maya Annie Elias, Manoj Kumar Pati, Praveenkumar Aivalli, C. M. Munegowda, Srinath Bhanuprakash, S. M. Sadhana, Bart Criel, Maryam Bigdeli, Narayanan Devadasan

**Affiliations:** 1 Institute of Public Health, 250, 2 c main, 2 c cross, Girinagar I phase, Bangalore, 560 085 Karnataka India; 2 Karnataka Health Systems Resource Centre, Leprosy hospital campus, Magadi Road, 1st cross, Bangalore, 560023 Karnataka India; 3 Institute of Tropical Medicine, Nationalestraat - 155, 2000 Antwerp, Belgium; 4 Alliance for Health Policy and Systems Research, World Health Organisation, Geneva, Switzerland

**Keywords:** Non-communicable diseases, Health systems research, Access to medicines, Out-of-pocket expenditure on medicines

## Abstract

**Background:**

India has the distinction of financing its healthcare mainly through out-of-pocket expenses by individual families contributing to catastrophic health expenditure and impoverishment. Nearly 70 % of the expenditure is on medicines purchased at private pharmacies. Patients with chronic ailments are especially affected, as they often need lifelong medicines. Over the past years in India, there have been several efforts to improve drug availability at government primary health centres. In this study, we aim to understand health system factors that affect utilisation and access to generic medicines for people with non-communicable diseases.

**Methods:**

This study aims to understand if (and how) a package of interventions targeting primary health centres and community participation platforms affect utilisation and access to generic medicines for people with non-communicable diseases in the current district context in India. This study will employ a quasi-experimental design and a qualitative theory-driven approach. PHCs will be randomly assigned to one of three arms of the intervention. In one arm, PHCs will receive inputs to optimise service delivery for non-communicable diseases, while the second arm will receive an additional package of interventions to strengthen community participation platforms for improving non-communicable disease care. The third arm will be the control. We will conduct household and facility surveys, before and after the intervention and will estimate the effect of the intervention by difference-in-difference analysis. Sample size for measuring effects was calculated based on obtaining at least 30 households for each primary health centre spread across three distance-based clusters. Primary outcomes include availability and utilisation of medicines at primary health centres and out-of-pocket expenditure for medicines by non-communicable disease households. Focus group discussions with patients and in-depth interviews with health workers will also be conducted. Qualitative and process documentation data will be used to explain how the intervention could have worked.

**Discussion:**

By taking into consideration several health system building blocks and trying to understand how they interact, our study aims to generate evidence for health planners on how to optimise health services to improve access to medicines.

**Trial registration:**

Protocol registered on Clinical Trials Registry of India with registration identifier number CTRI/2015/03/005640 on 17^th^ March 2015.

**Electronic supplementary material:**

The online version of this article (doi:10.1186/s12913-016-1680-3) contains supplementary material, which is available to authorized users.

## Background

### Access to medicines

The first point of contact for curative services in India is typically in the private sector, and often with medical practitioners without a formal medical degree in modern medicine [1–3]. In most Indian cities and in rural areas, private healthcare providers manage patients with chronic diseases deepening health insecurity for the poor [4, 5]. Even in cases where patients obtain outpatient care in government hospitals, they often have to purchase medicines from private pharmacies, either due to frequent stock-out of medicines at primary health centres (PHC), or poor procurement and distribution of drugs at higher levels [6, 7]. In spite of recent efforts through the National Rural Health Mission to improve utilisation of public services and decrease out-of-pocket expenditure (OOP) on medicines, recent estimates of OOP expenditure on medicines continues to be as high as 70 %, with instances of people falling into poverty merely from paying for outpatient care for non-communicable diseases (NCD) [8–10].

**Fig. 1:**
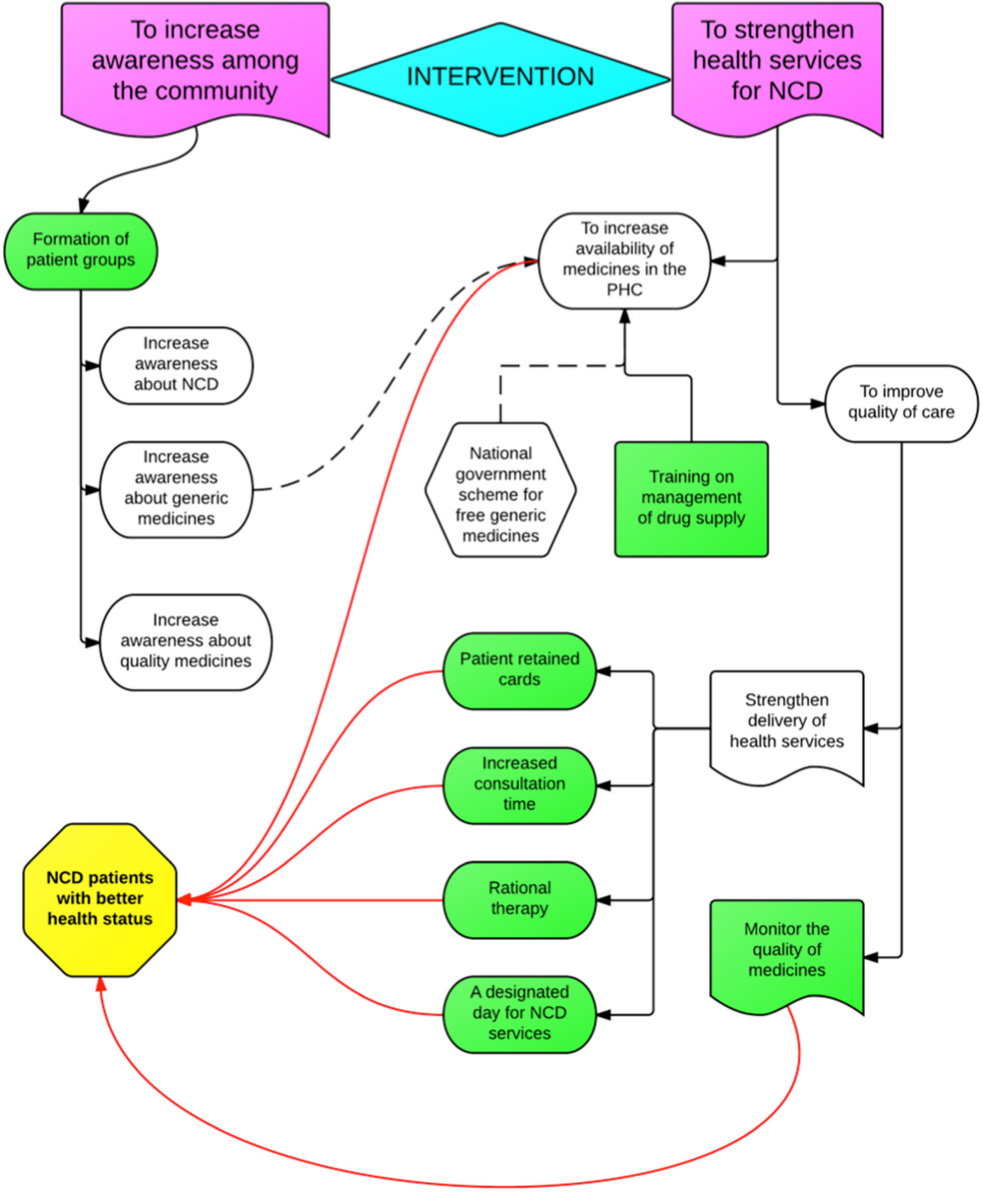
Problem tree illustrating government health services problems that could drive NCD patients to buy essential drugs from private pharmacies

The poor availability of drugs in public services is further complicated by inadequate availability of relatively inexpensive branded or unbranded generic alternatives in the private pharmacies [11]. In spite of India’s reputation as a global supplier of generic drugs, widespread reports of spurious medicines and the lack of an effective regulatory system for medicines affect people’s perceptions of quality of these drugs [12–14]. In addition, leadership and management of government health services at various levels within the health system influences access to medicines. The ways in which health systems organise their governance, resources use, organisation and management of services as well interact with people are being identified as the crucial in ensuring access to medicines, especially in public services [15] (see Fig. 1). Recent efforts at making available cheaper generic equivalents of essential drugs through *Jan Aushadhi* stores (people’s pharmacy in Hindi) have shown promising results; the generic drugs supplied have been reported to be comparable in quality to their proprietary equivalents [16, 17].

### Medicines and primary health care

Among the several approaches to organising care for NCDs in low- and middle-income countries (LMIC), the underlying principle is a well-performing primary health care system that ensures access to essential drugs for treatment of NCDs [18, 19]. Ensuring availability of medicines in government PHCs is necessary, but not sufficient to ensure access to medicines. Several factors ranging from individual and household perceptions, societal preferences and health-seeking behaviour to other factors related to health system, including availability, but also affordability, quality and management of government health services together characterise a well-performing health system. A systemic perspective on how these various factors interact is crucial in strengthening public services response to NCDs.

In this study, we aim to study the health system factors at the sub-district and PHC level that influence access to medicines for people with NCDs (diabetes and hypertension) in a rural Indian district setting. In doing so, we seek to understand how a mixed public-private health system, which is in the process of strengthening and decentralisation, such as the one in India responds to health services strengthening interventions to improve access to medicines.

### Rationale of the study: medicines and NCDs in India

Globally, there has been a lot of focus on the challenges in organising care for NCDs, especially in LMICs [18]. Recent publications highlight the inability of several LMIC health systems to deal with the rising needs of continuous care and follow-up required for patients with NCDs, as opposed to sporadic and episodic care for infectious diseases [20, 21]. There is increasing pressure on Indian policymakers at all levels to improve services for people with NCDs. Recently, a vertical programme, the *National programme for control of cancer*, *diabetes*, *cardiovascular diseases and stroke* (NPCDCS) was launched by the Indian government to address this challenge [22]. Various state governments in India as well as the national government have responded favourably through policies to improve access over the last decade. We take into consideration the following factors related to the policy environment within which this study is being proposed, thus of importance to the design of the study and, eventually for the interpretation of its results.

The governments of India and Karnataka have committed to investing more money in purchasing generic medicines and improving availability of these in government-funded/operated generic drug pharmacies [23, 24].

Recent reforms in Indian government health services through the National Health Mission (NHM) encourage community involvement in managing and financing primary health centres (PHC) through the establishment of *Arogya Raksha Samitis* (patient welfare committees). These committees have a mandate to oversee the PHC activities, as well as receive financial endowments from the State for improvements in PHC services.

## Methods

### Aim, objectives and research questions

#### Aim

The aim of the study is to understand health system factors for improving equitable access to quality generic medicines for patients suffering from non-communicable diseases in a rural Indian district.

#### Objectives

1. To understand if (and how) availability of drugs at government primary health centres (PHC) could be improved through formation of patient groups
2. To understand if (and how) utilisation, compliance and quality of care for NCD at government primary health centres could be improved through training PHC staff and optimising existing service-delivery arrangements.
3. To estimate the additional costs for increasing rational use of medicines and services optimisation at PHCs
4. To understand the health system factors that influence utilisation of drugs at government primary health centres
5. To document the effects on the private sector of improved drug availability and utilisation in PHCs

#### Research questions

The main research question of the study is: *does a package of interventions involving health services optimisation and strengthening community participation platforms at primary care level improve availability*, *affordability*, *utilisation and compliance of good quality generic drugs for NCDs in government facilities in Karnataka*? *If yes*; *how*, *and for whom and under what conditions does this happen*? We further developed five specific sub-questions in line with the main research question (and our study objectives listed above) as follows:

1. Does optimisation of health services (announcing monthly NCD clinic days, increasing consultation time, providing counselling services and ensuring continuity of care) improve utilisation of drugs and their compliance among patients with NCDs?
2. How can existing community participation arrangements be strengthened to form pressure groups of patients with chronic diseases at PHCs? Does this improve availability of drugs at these PHCs?
3. If NCD patients shift or opt for public sector generic drugs for NCD care, will their OOP expenditure on NCD medicines and proportion of total household expenditure (THE) spent on NCD drugs decrease?
4. Does the routine monitoring of the quality of NCD drugs strengthen the regulation of other drugs? How does the quality of drugs provided in government hospitals compare with those commonly bought over-the-counter in private pharmacies?
5. How do private pharmacies respond to improved generic drugs availability and utilisation in government PHCs? Do the mean prices of essential NCD drugs and their availability change in response to improved availability of these in PHCs?
6. What is the marginal cost of strengthening health systems so that access to medicines can be improved?

### Study design and conceptual framework

In this study, we use a quasi-experimental approach along with a theory-driven evaluation to study an intervention for improving access to medicines for diabetes and hypertension at PHCs in Tumkur district of Karnataka, India. We will use a quantitative before-after quasi-experimental study design to understand determinants of improved access to medicines, and a qualitative theory-driven evaluation approach to answer the *how* questions. All PHCs (39 PHCs) across three selected *talukas* were randomly assigned to one of three arms of the intervention. A baseline and an endline survey of households, PHCs and private pharmacies will be conducted for the before-after quasi-experimental component.

**Fig. 2:**
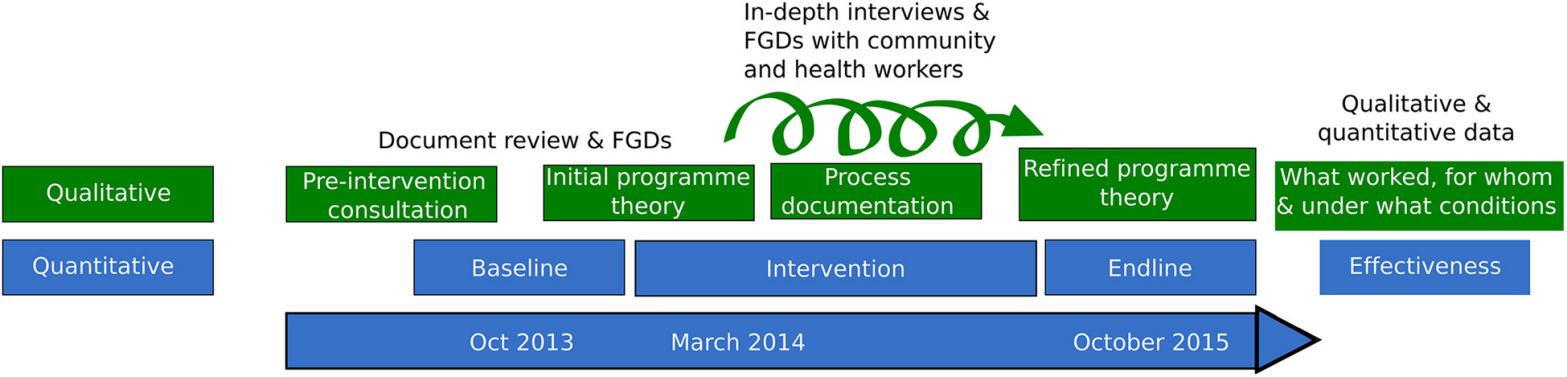
Study design of the Access to Medicines study

**Fig. 3:**
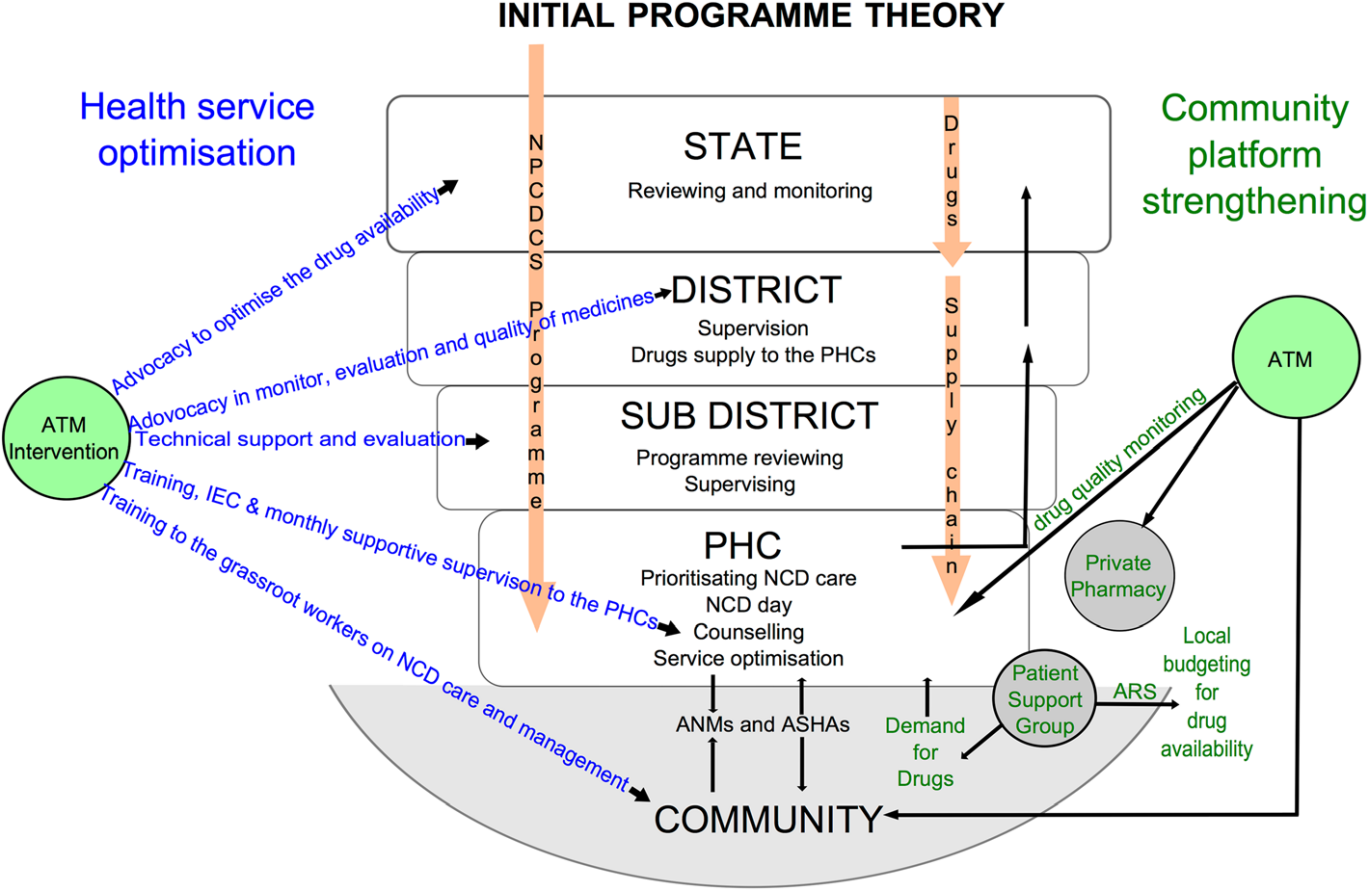
Initial Programme Theory

The qualitative component of the study will use a theory-driven approach to understand and explain the changes if any, and why the intervention worked for some (where it did) and not for others (where it did not) across the three arms of the study. The theory-driven approach uses successive waves of qualitative data to refine the programme theory, thus resulting in a contextual explanation for how the programme worked in the given setting [25]. In so doing, we shall generate an explanatory theory about improving access to medicines in local health systems in India especially focusing on what works, for whom, and under what conditions, and why. The study design and initial programme theory have been represented in Fig. 2 and Fig. 3. An initial programme theory is an explanation of why the programme is expected to work; it takes into consideration the inputs of the intervention and the assumptions of the implementers on how the intervention inputs could result in the expected outcomes, integrating contextual information from the implementation setting.

**Fig. 4:**
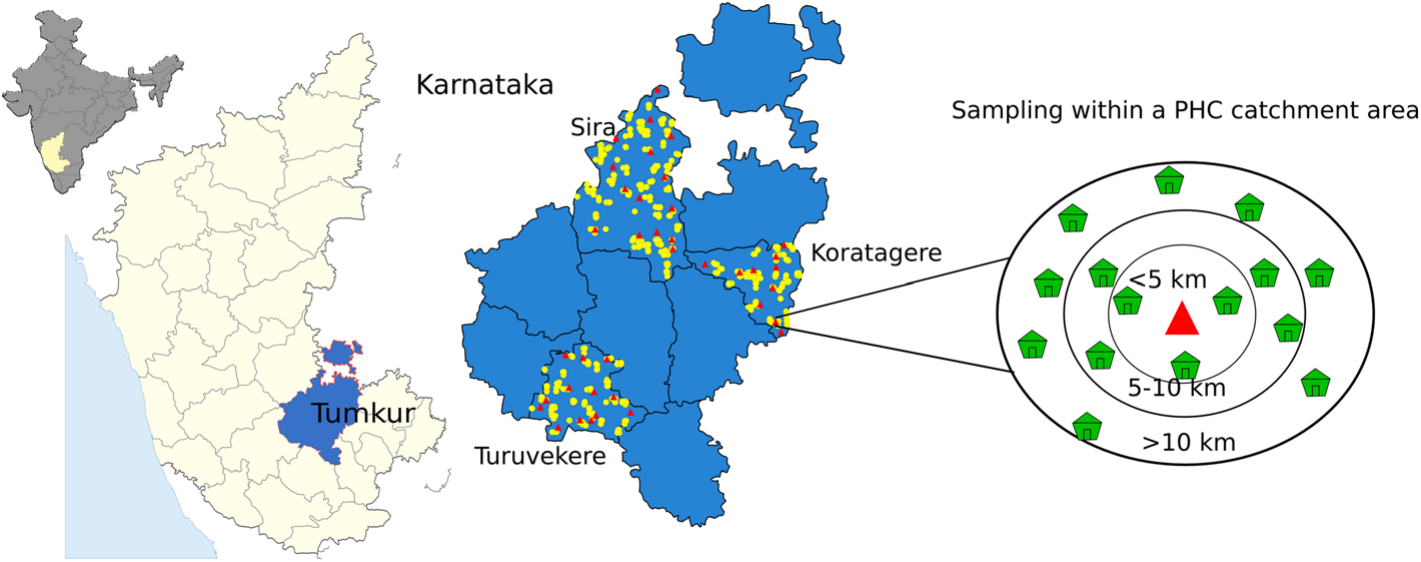
Study setting of the ATM project. Map by authors. Base map derived from Global Administrative Areas database (gadm.org) which is licensed for academic use with attribution

After the completion of the quantitative analysis (assessing effectiveness of the intervention) and the qualitative analysis (assessing the plausible reasons for the intervention working in some places and not in others), we will integrate the results into a final programme theory using the conceptual framework for access to medicines from a health system perspective proposed by Bigdeli et al. [26]. In Fig. 4, the Tumkur ATM intervention components have been represented in the Bigdeli et al. access to medicines framework. We will use this conceptual framework in explaining how the intervention contributed to improved NCD care in some settings, with a focus on the PHC and *taluka* level dynamic interactions between individual and institutional factors that contributed to the change, wherever it occurs.

#### Health systems interventions and complexity

Health systems interventions are implemented in existing, open complex adaptive systems [27–29]. This means that it is very difficult to expect a linear causal chain relating the inputs of the intervention to the proposed outcomes. Our proposed intervention shares some characteristics of a complex intervention:

1. **Path-dependence and resistance to change:** The mere availability of generic drugs in PHCs will not automatically change public prescriber behaviour or community behaviour of seeking more expensive branded drugs from private pharmacies. Changes in prescription practices as well as health seeking behaviour are crucial to improving access to drugs.
2. **Multiple interacting sub-components within open systems:** Health systems are characterised by several interacting elements that have a dynamic relationship amongst themselves [30, 31]. For example, community driven and/or socio-political factors could *push* the services into organising care for chronic diseases. While in one PHC, this could be due to community demands and power relationships between community representatives and the local staff, in another PHC, this could be due to the nature of the healthcare teams (their intrinsic motivation, leadership capacity etc.). The outcome we see could be determined by a dynamic interaction between several of these interacting sub-components of a local health system.

### Study setting

Tumkur district has 10 *talukas* with the population of these *talukas* ranging from 167,591 (Koratagere) to 596,000 people (Tumkur) [32]. A *taluka* is an administrative sub-unit of a district and consists of a hospital providing secondary care supporting a network of primary health centres (PHC), each PHC catering to a population of about 25,000 people. The *taluka* health office consists of a team of health managers led by the *taluka* health officer (THO), who is a doctor and other health workers. The team at the *taluka* level is expected to administer and supervise several PHCs and the *taluka* hospital, as well as implement various disease control programmes and other top-down schemes (such as the National Health Mission). In spite of several recommendations in favour of a dedicated public health cadre that is trained in public health management, most Indian states have not incorporated such a systematic public health management approach within their health services [33, 34]. The process of decentralised planning and decision-making is also in progress with the implementation of the NHM, India’s landmark health reform that began in 2005. It is within this macro-context that our intervention is being implemented in three *talukas* of Tumkur district. Maternal and child health programmes and other district interventions have been known to be influenced by the specific conditions in the local health system, as well as the nature of leadership and other characteristics of the management teams in these health systems [35].

The study is being implemented across the PHCs in three *talukas* (see Fig. 4) of Tumkur district of southern Karnataka. Tumkur is the second largest district in the state with an area of 10,598 square kilometres and has a population of 2.67 million of which around 30 % were in urban areas as of 2011 [32]. There is a mix of government and private sector, formal and informal providers as well as a range of single doctor clinics to secondary and tertiary level hospitals. The details of the facilities (both government and private) are given in Table 1. Despite significant achievements in providing and managing health services, inter-district and intra-district disparities in health outcomes persist in many Indian states [36, 37]. A recent government task force categorized Tumkur as an average district with respect to health outcomes and health services performance [38]. In terms of socioeconomic and development indicators, Tumkur could be classified as being one of the average performance district among the 30 districts of Karnataka state.

**Table 1:** Health services in Tumkur district [47]

Type of facility	Number
District hospital	1
Taluka hospitals	9
Community Health Centres	4
CHCs having functional OT	1
Primary Health Centres (24 × 7)	114 (44 % of all PHCs)
Sub Centres	477
Number of anganwadi centres	3378
Number of private hospitals in the district	106
Number of private clinics with allopathic doctors	96
Number of private hospitals performing LSCS	40

Each PHC has a doctor leading a team of nurses (at least 1), pharmacist (1), and few support staff with or without a laboratory technician. A PHC caters to around 20–40 villages with a population ranging from 25,000 to 30,000 people. For every 5–10 villages (approximately 3000 to 5000 population), a sub-centre with a nurse-midwife (Auxiliary nurse-midwife, ANM) caters to reproductive and child health services and a few other services under the disease-control programmes. A male health worker caters to one or more sub-centres. Under the NHM, a married woman of the village has been trained to function as a community health worker (Accredited Social Health Activist – ASHA). The ANM and ASHA are the main outreach staff that help in coordinating the many community-based activities of the PHC.

#### Rapid local health system assessment and choosing *talukas*

**Fig. 5:**
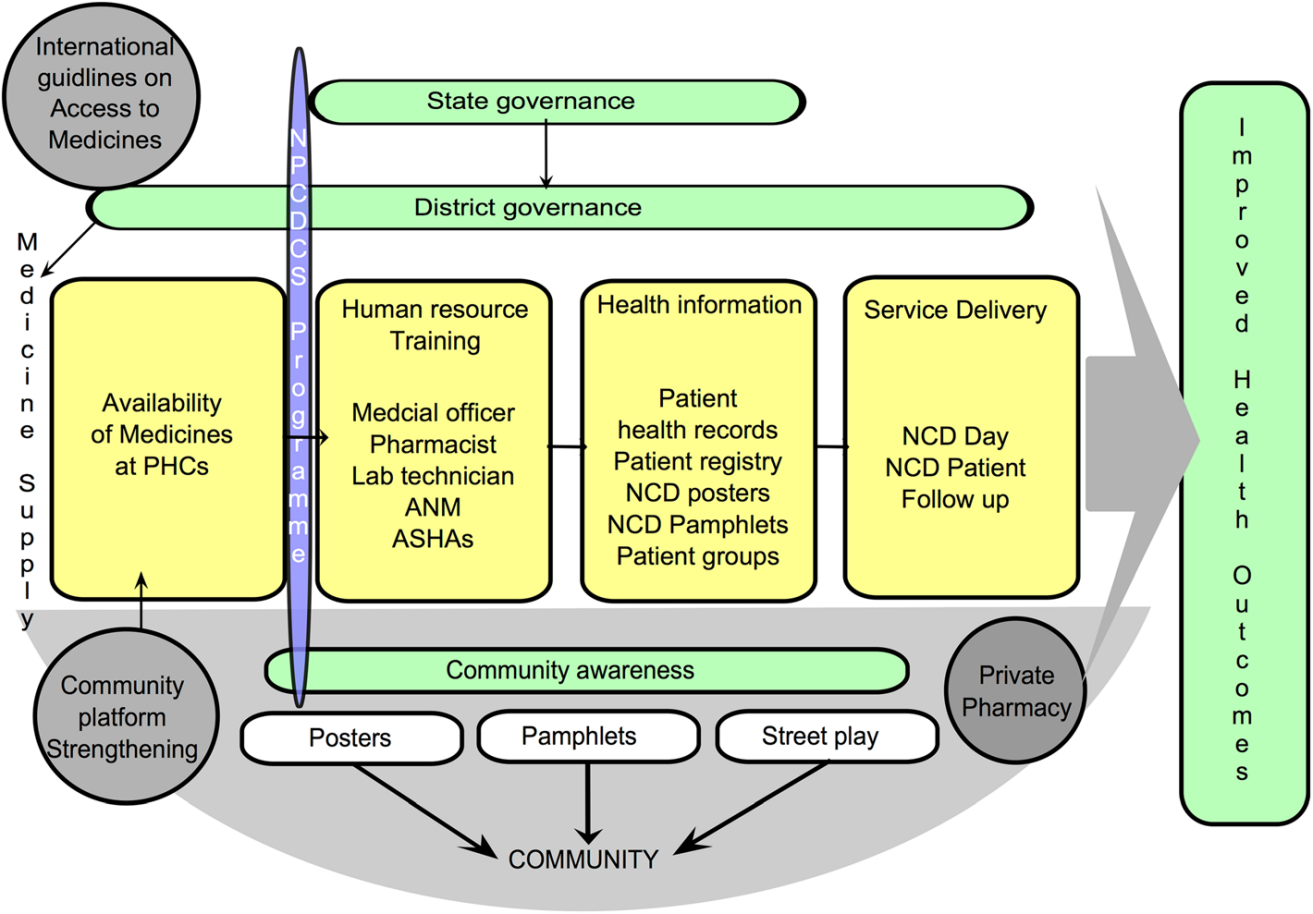
ATM conceptual framework

We conducted a rapid assessment of the performance of the local health system with respect to its various building blocks, following the health systems dynamics framework and the Bigdeli et al. framework (see Fig. 5) [26, 30]. The objective of the assessment was to exclude *talukas*, which do not have the basic conditions needed for a medicines-related health systems intervention such as the one being implemented. We used the framework to identify various proxy indicators of performance of particular health system domains and excluded talukas that performed poorly with respect to the central axis of the health systems: governance, human resources, service delivery and population. Three *talukas* were excluded for not having the necessary system preparedness for the intervention based on this assessment. The full details of the process of the rapid local health system assessment are included in Additional file 1.

**Fig. 6:**
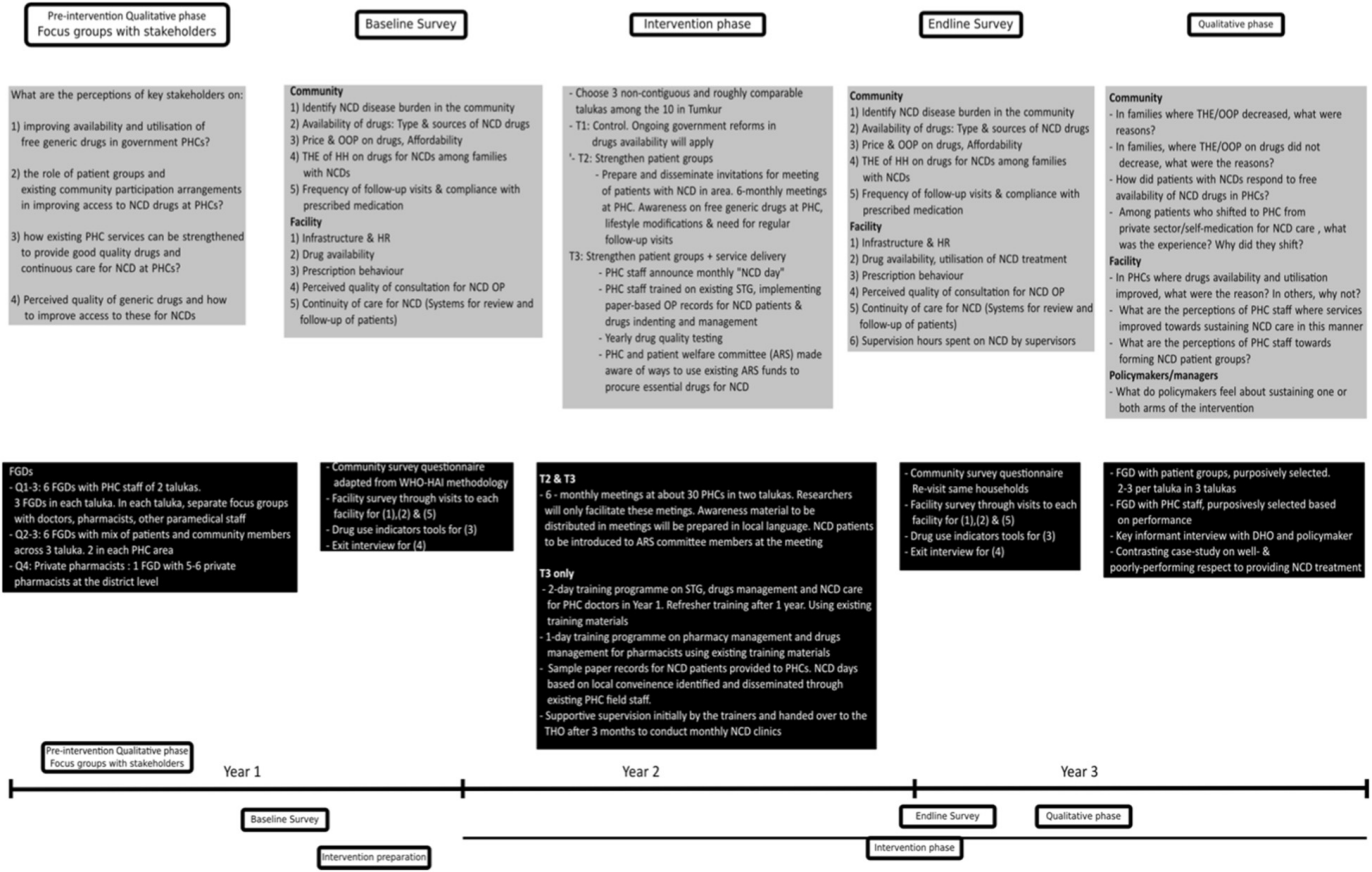
Step-wise rolling out of the study intervention

Of the seven *talukas* which qualified for the intervention, we randomly chose three *talukas* using the online random number generating tool (Available at http://www.random.org/; see Additional file 2). The three *talukas* chosen were Koratagere, Sira and Turuvekere. Some characteristics of the three *talukas* are presented in Table 2. PHCs across the three *talukas* were randomly assigned to one of three arms (A, B and C) of the study intervention. In Arm A, PHCs will receive a package of interventions to optimise service delivery, while Arm B, they will receive Arm A package as well as an additional package of interventions to strengthen existing community participation platforms for organising NCD care at PHC. PHCs in Arm C will be the control. The package of activities in A, B and C are described in Table 3. A detailed description of the intervention and the underlying assumptions behind the activities proposed is provided in Additional file 3 and step-wise plan of rolling out of the intervention is presented in Fig. 6.

**Table 2:** Characteristics of study talukas

Characteristics	Koratagere	Sira	Turuvekere
Area	652 Sq km	1552 Sq km	778 Sq km
Population	160,952	301,473	174,297
Number of private pharmacies	19	30	22
General adult literacy rate	71 %	67 %	73 %
Number of PHCs	11	17	11
Average population per PHC	14,500	18,000	16,000
Number of private hospitals/clinics	21	64	18
Number of PHCs with qualified MBBS doctors (of) total number of PHCs in *taluka* (as on March 2014)	11 of 11	15 of 17	10 of 11

(Source: Census 2011)

**Table 3:** Description of activities planned under the study intervention arms

Intervention arm	Intervention name	Intervention input	Activities
A	Optimise service delivery	Orientation training & workshop for PHC medical officers	Training on standard treatment guidelines for NCDs, rational prescription, maintaining treatment cards for patients with NCDs and conduction of health days for NCD care.
		Training workshop for pharmacists	Training on need assessment for medicines, counselling patients on medicines and their side effects and non- drug treatment, indenting for medicines and record keeping at PHCs.
		Training workshop for ANMs, ASHAs and Anganwadi workers.	Orientation on lifestyle modification for NCD, need for long-term follow-up and medication for NCD, availability of free generic medicines for NCD at local PHCs and ensuring that patients visit the PHC regularly for follow-up and medicines.
		Advocacy and coordination.	Coordination and advocacy at state, district and taluka levels with different stakeholders to ensure supply of drugs to the PHCs through routine supply chain, district PIP or utilising local funds (ARS).
B	(Optimise service delivery) + Strengthen existing community participation through patient groups and ARS	Development and dissemination of awareness material.	Awareness material shall focus on lifestyle modification for NCD, need for long-term follow-up and medication for NCD, and availability of free generic medicines for NCD at local PHCs. Disseminate material among health workers, local community groups (ARS, VHSC).
		Formation of NCD patient groups.	To organise NCD patients group meeting and inform about the importance of regular treatment and the advantages of generic medicines
		BCC at community level.	Health worker spreading information leaflets about diabetes & hypertension, about the designated NCD check up day event held 1–3 times per year at the PHC. Information on NCD control will also be displayed at prominent places in the PHC and local villages
		Meeting with ARS members.	Orientation of ARS members about their functions and possibility of utilizing untied funds for purchasing medicines for NCD and facilitate their interaction with patient groups and PHC staff
C (Control)	No intervention	Government existing programme for NCD control at primary health centres.	Government’s making effort at improving generic drug availability. Private pharmacies cater to many NCD patients.

### Data collection and tools

**Fig. 7:**
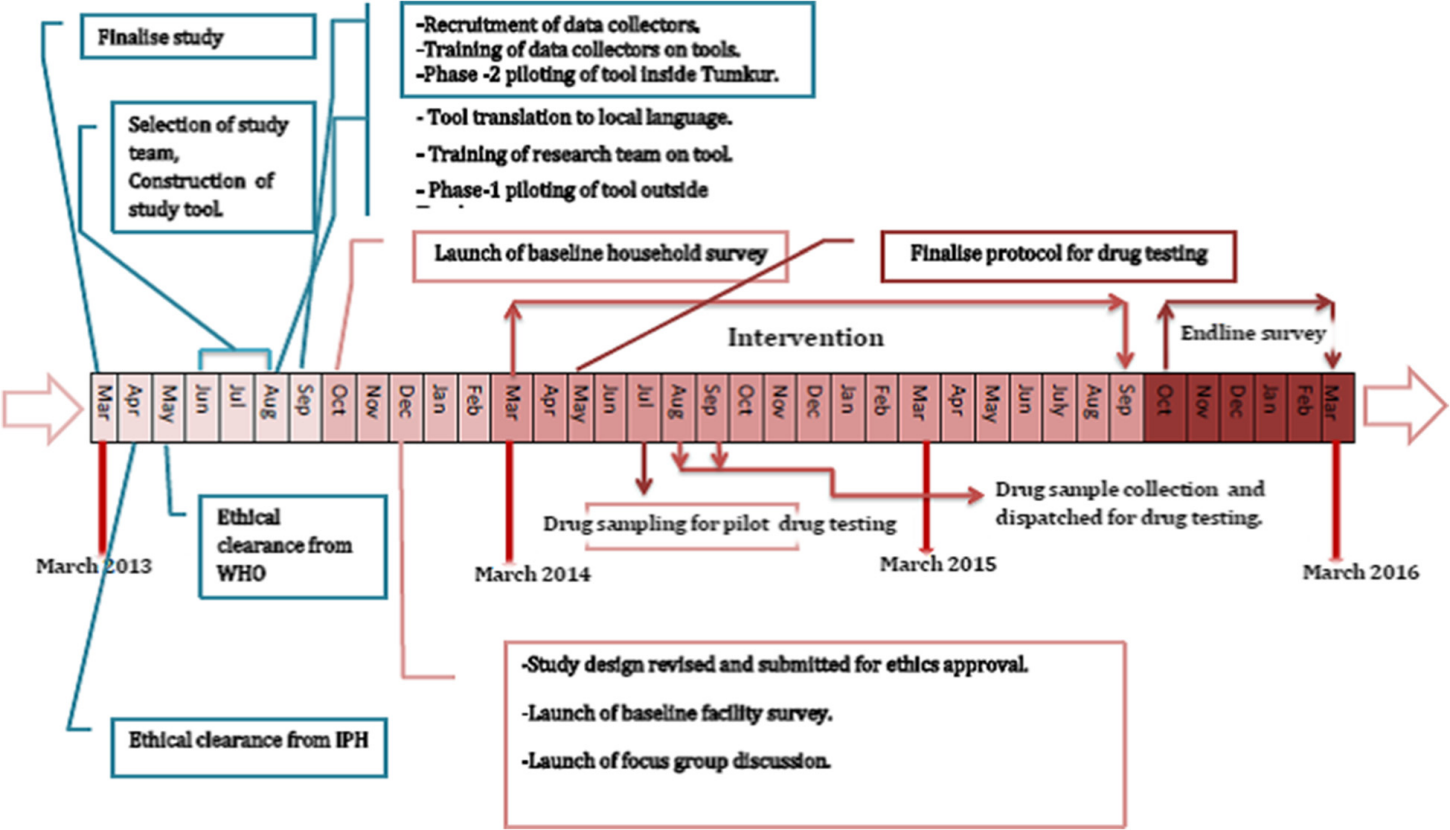
Timeline of the Tumkur ATM study

The study tools, study preparation, process of data collection are described below (See also Fig. 7 on the study timeline). The quantitative data collection includes household surveys, facility surveys (public and private pharmacies) and exit interviews at PHCs. The qualitative component includes focus group discussions (FGD) with district-level officials, PHC staff and patient groups.

#### Quantitative data and tools

##### Household survey tool

The household survey tool was based on the standardised WHO tool to conduct household survey and measure access and use of medicines based on a standardised WHO tool included in the WHO Operational Packages for Monitoring and Assessing Country Pharmaceutical Situations (World Health Organization [39]; see Additional file 4). The tool measures basic demographic and household characteristics, people’s health seeking behaviour, availability of medicines at homes, cost, affordability and appropriate usage of medicines by the NCD patients in these households. The questionnaire was adapted to suit the local context by choosing modules relevant to our study as well as adapting it for NCDs (the tool is originally designed for communicable diseases). Our final tool was divided into six parts to collect routine demographic information, health seeking behaviour, socio-economic status, perceptions on price and quality of NCD drugs and access to and use of medicines for NCD. We added additional questions to collect data on utilisation of PHC services for NCD, out-of-pocket (OOP) expenditure on NCD drugs, proportion of total health expenditure (THE) on NCD drugs, compliance with medication and awareness on generic drug availability at PHC.

##### Facility survey tool

For the facility level survey, we used a modified version of Level II survey tool included in the WHO Operational Packages for Monitoring and Assessing Country Pharmaceutical Situations (World Health Organization [39]; see Additional file 5). This tool shall be used to collect data from PHCs, private pharmacies and the district medicines warehouse. For the facility survey tool, we used the Karnataka state standard treatment guidelines to finalise a list of medicines that are supposed to be used for NCDs at PHC level. 11 medicines were included in the survey tool.

The facility survey will assess various aspects of NCD drugs such as the availability of key essential medicines for diabetes and hypertension, their stock-out duration, record-keeping at the facility, conservation conditions and handling of medicines, patient care and prescribing practices, price, storing, availability of standard treatment guidelines and essential medicines list.

##### Exit interview tool

The exit interview tool will collect data about average number of dispensed medicines per prescriptions and the travel cost borne by NCD patients (see Additional file 6). The tool will assess how many of the prescribed medicines were dispensed at the PHC and if the medicines were adequately labelled (the label should contain the medicine’s name and dosage), if the patients knew about the dosage and duration of all dispensed medicines, and how much the patient paid out-of-pocket for medicines and/or in non-diagnostic fees.

This tool was translated to the local language Kannada and was verified by a native speaker of Kannada. The tool was further modified based on two phases of piloting of the tool.

#### Training of team members and piloting of tool

The first phase of piloting was conducted at two PHCs outside in a district adjacent to Tumkur. This phase focused on the way the survey respondents interpreted the local language questions and the quality of the data. After the first round of piloting, we invited an external expert who was familiar with the application of this tool in Indian settings. We conducted a training workshop to train all the data collectors in administering the tool. After the workshop, the expert and the team organized a second phase of piloting to ensure that the modified tool is technically sound and to consider any new ethical implications for the changes made. For the second phase, the data collectors visited a PHC in the neighbouring district under supervision of the co-investigator. All issues and challenges faced with the interviews both at household, public and private healthcare facilities were discussed at length and the tool refined further.

The tool was also presented and discussed with key actors in state government and the district health team, and their permission was sought to conduct facility surveys in the PHCs.

### Survey sampling

#### Quantitative data

##### Household survey

The WHO household survey tool does not have inclusion criteria related to NCD. Sample size calculation was based on obtaining at least 30 households per PHC spread across three clusters (a, b and c with 10 households per cluster; see above). A typical PHC in Tumkur district has at least five villages in each cluster, and hence at least 1000 households in each cluster (approximately 250 households in a village). We aimed for a total of 1170 households anticipating 30 households per PHCs across the 39 PHCs.

We started the survey at the PHC where we listed all the villages under the PHC area. These villages were then divided into the three clusters based on the distance from the PHC and from each cluster, one village was randomly chosen. We randomly selected a minimum of 10 NCD households from all four directions starting from the central point of the village. After getting informed consent of the participant the survey questionnaire was administered to the NCD patient for the interview. In his/her absence, the spouse or another member of the household who was over 18 years of age, aware of the information sought in the survey and willing to provide this information was interviewed. The data collectors started the survey within each village in four different directions and ensured that all types of houses and areas within the village were included in the sampling. Wherever 10 NCD households were not obtained in one village, the data collectors repeated the process in the nearest adjoining village. Each participant was provided with an information sheet and health awareness material in local language related to NCDs (Additional file 7) as well as contact information of the research team.

We surveyed 10 households from within five kilometre radius from the PHC (cluster a), 10 from between five to 10 km radius (cluster b), and 10 from more than 10 km distance from the PHC (cluster c). All of the 39 PHCs in these three *talukas* were covered, resulting in 1069 NCD households surveyed as presented in Table 4 and Table 5. The same households shall be sampled during the endline survey planned in last quarter of 2015.

**Table 4:** Distribution of households participating in the study by *taluka*

S No.	Taluka	Number of households interviewed
1	Turuvekere	326
2	Sira	482
3	Koratagere	261
Total		1069

**Table 5:** Number of PHCs covered in the ATM study talukas

Taluka	Number of PHCs
Turuvekere	11
Sira	17
Koratagere	11
Total	39

**Fig. 8:**
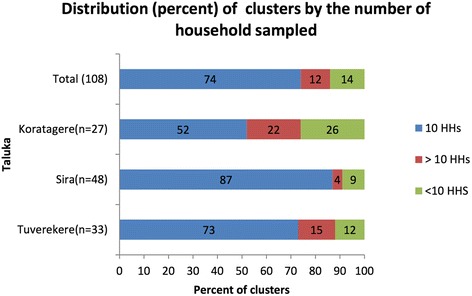
Percentage of clusters across study talukas showing sampling adequacy

**Fig. 9:**
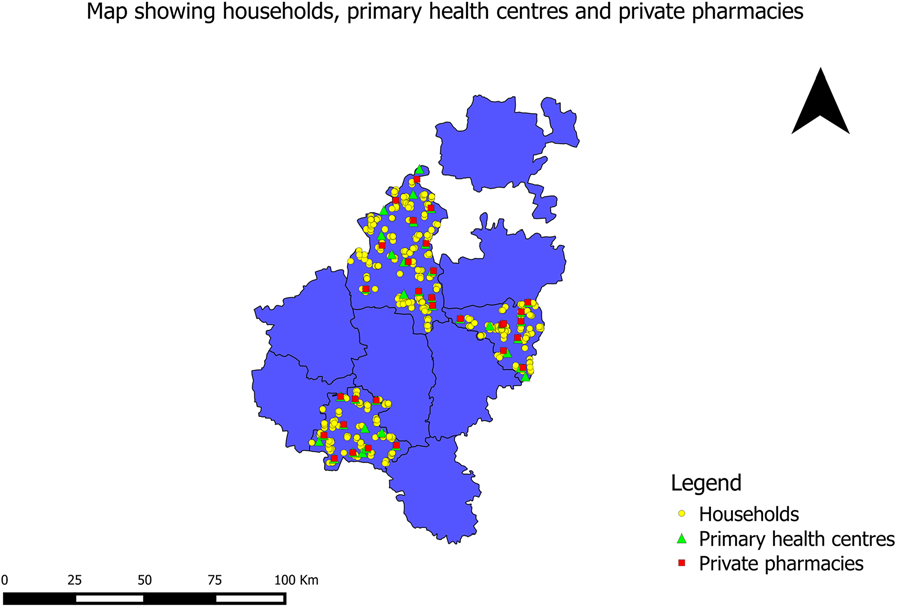
Map showing all the sampling points (households, PHC and private pharmacies) in the Tumkur ATM study. Base map derived from Global Administrative Areas database (gadm.org) which is licensed for academic use with attribution

**Fig. 10:**
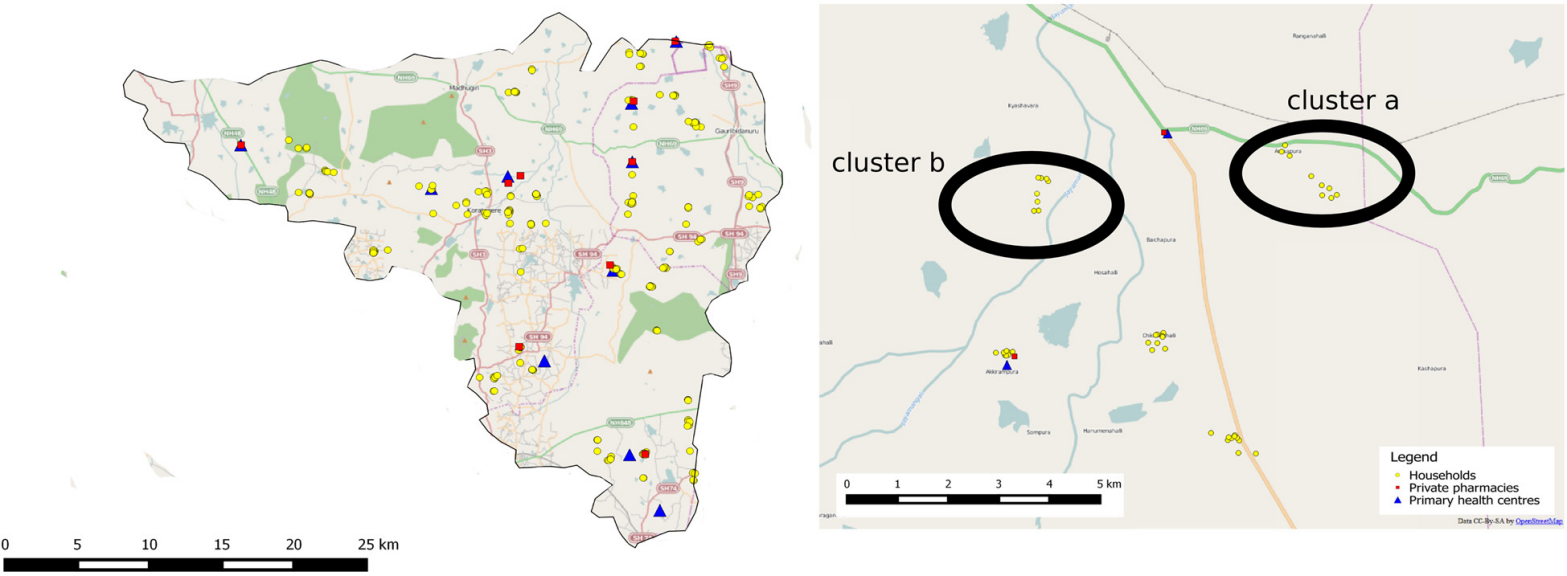
Map showing sampling of households and facilities in Koratagere taluka. In some PHCs with smaller area of coverage, only two distance-based clusters were identified for the household survey. Base map derived from Open Street Maps layer which is licensed under creative commons license

**Fig. 11:**
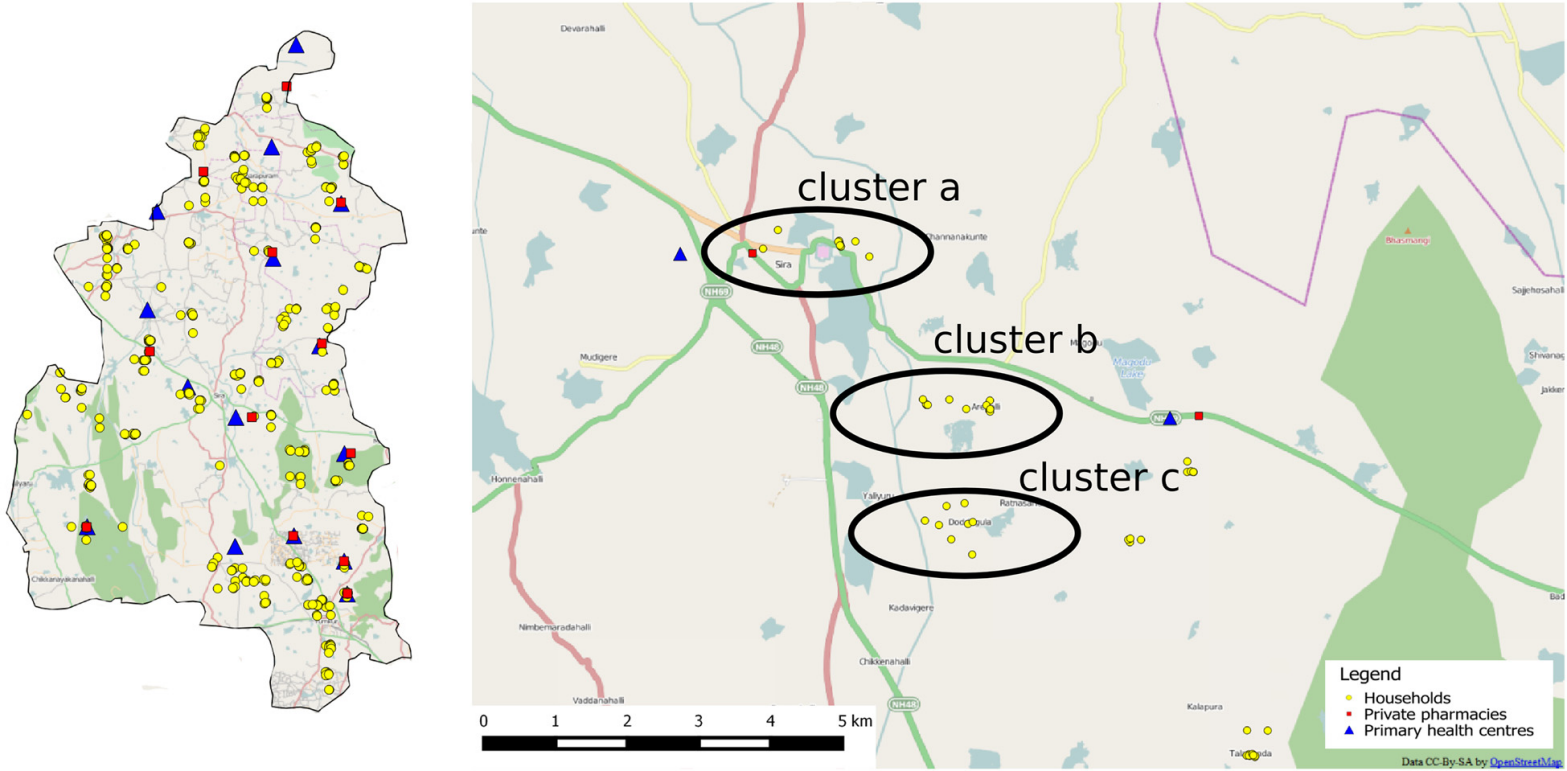
Map showing sampling of households and facilities in Sira taluka. The three distance-based clusters are highlighted. Base map derived from Open Street Maps layer which is licensed under creative commons license

Geographic coordinates were captured for each of the household and facility surveyed using a hand-held global positioning system (GPS) device (GARMIN model EPX 300). This will enable generation of maps to summarise the household and facility data using QGIS, a geographic information system application that allows for geo-spatial data analysis and mapping (QGIS Version 2.4). There were PHCs where we could not sample cluster b and/or cluster c due to their small geographic spread (see Table 6). In other instances, we sampled sometimes in excess and sometimes lesser than desired number of households based on the availability of a NCD patients in these houses (see Fig. 8). The various households and facilities (PHCs and private) where data was collected are represented in Fig. 9. In some instances, where areas covered by a given PHC were smaller, we could only identify two distance-based clusters (a and b), as seen in Koratagere in Fig. 10. Similarly, the sampled households in Sira are shown in Fig. 11.

**Table 6:** Distribution of sampled clusters (a, b and c) across the ATM study *talukas*

Taluka	Number of cluster a (<5kms)	Number of cluster b (5–10 km)	Number of cluster c (>10 km)	Total
Turuvekere	11	11	11	33
Sira	17	17	14	48
Koratagere	11	10	6	27
Total	39	38	31	108

The survey form typically took an about 30 min to administer, and an average of ten questionnaires was given per field investigator per day. The filled-in forms were checked for completeness and appropriateness of responses by a supervisor the same day. In case of incomplete forms, the field investigator revisited the households. To validate the data collection the supervisors randomly picked one survey form in every 10 forms and visited the household to check the validity of the data. The supervisors were provided with structured checklist (Additional file 8) to ensure completeness of the survey forms. During the weekly meetings, the study team reviewed all the collected forms in that particular week, and discussed any challenges and sought information on any potential ethical issues.

##### Facility survey

For the facility survey, we sampled from the district drug warehouse (*n* = 1), all the PHCs (*n* = 39) and the private pharmacy nearest to the PHC (*n* = 30) (Table 7). At the private pharmacy, we collected data on the *most sold* and *least priced* generic medicines for NCDs along with storage conditions and price of the medicines. Consent was obtained to collect information at each facility; an information sheet was provided to the pharmacist at each facility. The stock-out duration and the indenting price (the cost price) were physically verified by the research officers in all the facilities.

**Table 7:** Distribution private pharmacies interviewed across the study talukas

S No.	Taluka	Number of private pharmacists interviewed
1	Turuvekere	9
2	Sira	12
3	Koratagere	9
Total		30

#### Exit interviews

A trained data collector conducted exit interviews. At each PHC, the data collector recruited NCD patients over a period of three days per PHC for all the PHCs (*n* = 39). Each patient exiting the facility was approached. If the current consultation was for Diabetes/Hypertension, then the study details was explained and written consent obtained to conduct the interview. The target was to interview 30 patients but we were able to conduct, on an average, eight interviews per PHC over the three days. A daily debrief was organised by the researchers to identify and respond to any ethical or operational challenges in course of the day.

##### End-line survey

After 18 months of the intervention, the survey will be conducted in the same three talukas. The same households (panel survey) that participated in the baseline survey will be visited again with the survey questionnaire.

#### Qualitative data

Qualitative data will be collected in two phases; it will be used on one hand to guide the contextualisation of the intervention, and on the other hand for a process evaluation of the intervention.

Initially, we used data from focus group discussions and in-depth interviews with communities and health managers to contextualise the implementation of the intervention. The data was also used to prepare an initial programme theory (how is the intervention supposed to work). After the implementation of the intervention, we will use qualitative data to understand why it worked in some places (where it did) and not in others (where it did not) through the refining of the initial programme theory. The refining of the initial programme theory involves critically assessing the assumptions of the initial programme theory in relation data collected during the intervention implementation as well as an understanding of how such interventions are supposed to work in wider literature. Programme theories can be progressively refined as the intervention is implemented and monitored. The refined theories generated could be used to understand the relationships between intervention inputs, local context and the outcomes seen [29, 35, 40].

For developing the initial programme theory, we will conduct a content analysis of relevant documents including project documents, documents and reports of the National Programme for Control of Diabetes Cardiovascular diseases and Stroke, the recently launched national programme on NCDs and various pharmaceutical policies at state and national level. The content analysis will be used along with review of literature and the baseline qualitative data to build an initial programme theory on why the intervention is *supposed* to work and what possible local factors could be critical for the implementation of NCD care at PHCs.

In-depth interviews, process documentation during the implementation of the intervention and another round of qualitative data collection through focus group discussions and in-depth interviews at the end of the intervention period will be used to refine the initial programme theory and understand the health system factors that could have contributed to positive outcomes in PHCs where it did work, and the possible contribution of the intervention.

##### Focus group discussions

As part of the baseline activities, we conducted focus group discussions (FGD) with various stakeholders such as community members, health workers, government doctors (PHC medical officers) and pharmacists in private pharmacies. A total of 13 FGDs were conducted. Each FGD had about 8–10 participants.

The FGDs among the community members were conducted with an objective of understanding the experience and identifying barriers in accessing care at PHCs, more so for patients with NCDs. We in fact tried getting different perspectives by conducting these FGDs among diverse groups i.e. men, women, NCD patients, healthy adults, and people from different socio-economic categories. The health workers FGD consisted of Accredited Social Health Activist (ASHAs) and Auxiliary Nurse Midwife (ANMs). The discussions with the PHC medical officers were conducted separately. These FGDs focused on PHC doctors’ experiences and challenges in delivering care for patients suffering from NCDs at primary care level, including pharmaceutical and behavioural management. Their perspectives on access to medicines in general and on services delivered at the PHC were also documented. We invited pharmacists who owned or operated private pharmacies in Tumkur town to participate in an FGD. In this FGD, we explored their perceptions on price and quality of the medicines supplied both in government and private pharmacies and also on their view on generic medicines.

All FGDs were audio-taped and transcribed. The participation in the FGDs was voluntary and no compensation was provided to the participants. Informed written consent was obtained from all FGD participants. All participants were given written information about the project. The details of the FGDs conducted in the baseline phase are in Table 8.

**Table 8:** Details of focus group discussions conducted in the baseline phase

S No.	Number	Group	Content	Target population
1	2	Patients	NCD burden & care	NCD patients
2	2	Healthy adults (Non NCD)	Quality of health care	Non NCD patients & other healthy individuals
3	4	Health workers	NCD burden & community awareness	ASHAs and ANMs
4	1	Private drug shopkeepers	Quality generics for NCD	Tumkur district private drug shopkeepers

##### In-depth interviews

In-depth interviews were conducted with various actors in the heath system to understand their perspectives on different issues around NCD management and access to medicines. Suggestions were sought from them on the design and implementation of the study intervention to improve access to medicines for NCD patients. The feasibility of the intervention was discussed and the suggested modifications were incorporated.

#### Process documentation

Process documentation forms an integral part of the qualitative component of this study. Quarterly field visits will be conducted to the PHCs and data on utilisation of various services and medicines dispensed for NCDs will be recorded. The patient experiences, PHC staff attitudes towards NCD patients and details of supervisory visits from taluka health officials would be captured. The latter is in order to understand and interpret possibly taluka-wise differences in local leadership and management that could explain differences in performance between PHCs that have received similar inputs.

The interaction with the providers would focus on the motivating factors in case of early adopters and the perceived difficulty in case of others. After each such visit, a detailed narrative report would be prepared. This report would capture unstructured details about: new changes or updates regarding NCD patients in the PHC, responses of PHC staff to the intervention-related developments such as PHC meetings, sub-centre meetings and PHC health days (in the intervention PHCs). In the control PHCs, the field visits will be used to collect new information on any initiatives to detect or treat NCD patients and perspectives on the staff on NCD care.

As part of the community level intervention, we will participate in the patient group meetings and observe whether the group dynamics and the interaction of the group with the PHC have changed/improved, this will be noted and these groups will be purposively approached during the post-intervention data collection.

#### Drug quality test

A quality test for NCD medicines has been planned as an independent activity of the study. Of the 11 drugs included in the facility survey, four were chosen (two anti-diabetic and two anti-hypertensive medicines) for quality testing. Generic and branded medicines were sampled from both government and private facilities in the three study talukas, Tumkur district as well as the nearest big city, Bangalore. The full details of the sampling and the tests are in Additional file 9.

### Data analysis

The quantitative and qualitative analysis will first be done independent of each other. At the end of the baseline survey and during process documentation, the qualitative data will be analysed iteratively for refining the programme theory of the intervention.

#### Data entry and cleaning

The original household survey data were labelled and stored so that researchers can return to the original source of the data if necessary. A data entry form with inbuilt restrictions and validity checks for the fields was created using Epidata [41]. In addition, 10 % of the data entered randomly were verified with the survey questionnaire by a supervisor. Any discrepancy (missing or illogical values) was corrected by checking the paper questionnaire and if any crucial missing data, field visits may be made again, if necessary.

#### Quantitative data analysis

The quantitative data will be transferred to data analysis software (SPSS) and basic variables will be created. All the codes and value labels of the variables will be recorded and saved for further use if any. We will create three datasets from the household survey to capture basic household, demographic and patient (NCD) level characteristics. Then, these datasets will be merged and the merged dataset will be independently validated through running similar queries on at least two different statistical analysis software (SPSS and R). Private facility interview data and PHC interview data will be merged after cleaning. Exit interview data will be also be cleaned and analysed separately.

#### Univariate analysis

Basic tables and graphs depicting summary statistics of the respondents (individuals, households and patients) will be done– example percentage of women, age distribution, caste, religion, type of households, household amenities, number of diabetics and hypertensive, availability of medicines in the house and health facilities etc.

#### Bivariate analysis

We will check for association (Chi-square distribution) and linear and partial correlations among selected variables in line with the hypotheses/research questions identified in the research protocol. Baseline and end-line household and facility level characteristics will be compared across the intervention arms and study talukas.

#### Intention-to-treat analysis

We will conduct an intention to treat analysis at the PHC level. Independent variables such as socio-demographic characteristics will be compared to assess comparability across intervention arms, using relative risk ratios at baseline and endline. Like in the cases of classical trial studies, when the unit of the intervention (PHCs in this study) does not follow the protocol for their assigned treatment/ intervention, the resultant “treatment contamination” can produce misleading findings. Intention to treat analysis will be used in this case to estimate the effect of recommending a treatment to study participants, not the effect of receiving the treatment. Further a technique called *contamination adjusted intention to treat analysis* will be attempted to complement the intention to treat approach by producing a better estimate of the benefits and harms of receiving a treatment/intervention [42].

#### Subgroup analysis

We will analyse various sub-groups like gender, age, caste, socio-economic category, distance of household from PHC, in relation to their access to medicines, affordability, or availability of key medicines for previous six months. We will compare these sub-group characteristics across intervention arms.

#### Covariate-adjusted analyses

This analysis will aim at establishing the improvements (in terms of outcome variables) due to the intervention. We will take into account the non-equivalence of the three intervention groups with respect to some baseline characteristics. This will be examined through logistic regression. Robust standard errors will be adjusted for clustering at village level. We will check and compare adjusted and unadjusted odds ratio while doing the logistic regression.

#### Asset index

An asset index will be constructed for the households by performing principal component analysis on key household characteristics like household ownership of house, a two wheeler and domestic animals, utilities used in the household like tap water inside their houses, access to electricity or access to other amenities [43, 44].

#### Difference in difference analysis

Difference in difference estimation will be used to test the effect of the intervention on the outputs. This analysis will adjust for the possibility of other factors like time, institutional factors leading to the output.

#### Cost-effectiveness analysis

Finally we will analyse the cost data on the expenditure on the various intervention inputs consisting of human resources, training, community mobilization, awareness promotion materials, stationary and overheads. These costs will be compared with the outcomes (availability of key generic NCD medicines in PHC, prescription of generic medicines, regular and rational consumption of medicines among NCD patients) extrapolated at the population (NCD) level for the study arms. Incremental cost effective ratios will be estimated against the control arm.

#### Outcome estimation

Outcomes (see Table 9) will be reported as effects of said interventions and represented as risk ratios and odds ratios with 95 % confidence interval.

**Table 9:** Measures (indicators) for primary and secondary outcome of interest

Factors to be measured	Indicator
Primary outcome of interest	
Improved and continuous availability of generic drugs at PHC	Percentage of PHC where all generic medicines for NCD patients adequately available in last three months
Increased and regular access to quality medicines for patients with NCD	Percentage of NCD patients/patient groups visit intervention PHC in last three months
Out-of-pocket expenses among patients with NCD	Percentage reduction in median OOP expenditure on medicines per year among NCD patients.
The marginal cost of the intervention to that of the outcome	Incremental cost effective ratio
Improved compliance to standard treatment guidelines by the PHC	Percentage MOs followed prescription practices as per the standard treatment guidelines.
Improved demand of generic drugs among the patients	Percentage increase in patients availing generic drugs from the PHC compared to what it was at three months back
Improved utilization of medicines among NCD patients	Percentage of NCD patients/ patient groups obtained complete and regular medication for last three months.
Secondary outcome of interest	
Better coordination among health staffs with regards to NCD patient group formation	- Percentage increase in number of patient groups formed. - Percentage increase in number of new diabetes patients registered in last three months for a given PHC. - Percentage increase in number of new hypertension patients registered in last three months for a given PHC.
Better drug procurement	Percentage PHCs where drug procurement is up to date for the last quarter.
Conduction of NCD clinics	Percentage PHCs conduct NCD days in last three months
Better reporting and recording by the pharmacists at PHC	Percentage PHCs where medicines were dispensed as prescribed

#### Qualitative data analysis

All the process documentation data and the baseline and endline interview transcripts will be managed through uploading in a secure and password-protected account in Evernote, a cloud-based data storage software that allows for tagging and organising various forms of data automatically, for place, type of data and coordinates [45]. This data will be directly imported into NVivo for coding.

The qualitative data analysis will draw from the principles of theory-driven approach, in this case starting with the initial programme theory. We will analyse the qualitative data by examining the extent to which the intervention designers’ assumptions were borne out, especially looking for alignments as well as contradictions to these programme theory assumptions. In doing so, we will borrow from wider literature on how and why interventions such as this one have been studied in literature, as well as scanning our data for micro and meso level contextual factors that affected programme performance and/or outcomes [26]. We will thus refine the initial programme theory enriching it with the contextual understanding of the intervention in order to explain how the intervention worked (where it did) and under what conditions it worked. The resulting refined programme theory at the end of the intervention will be useful in understanding how local health systems such as Tumkur respond to resources that such interventions provide.

## Discussion

### Understanding the *why* and *how* of interventions

Healthcare organisations and systems are complex entities, which do not respond uniformly and predictably to a given policy. Feedback loops and path dependence often result in policy failures or inadequate response, while there could also be unintended and unforeseen consequences of interventions. We have developed an intervention based on the gaps in the present health system that need to be addressed for the above policies to result in improved access to medicines for people with NCDs.

Our intervention is being implemented at the district level in partnership with the district health services. It could be described as a complex intervention in view of the involvement of actors at various levels of the health system with varying interests, powers and relationships. Innovative health services interventions need to be designed in LMICs to better understand the conditions that could enable improvement in complex health systems. Hence, integrating experimental study designs (effectiveness) with qualitative methods (process) is crucial. Our study provides a model for strengthening health services for NCDs and identifying the necessary conditions for improvements. By taking into consideration several components of the health system (governance, service delivery, resource planning and community) and trying to understand them, our study aims to generate evidence for health planners on how to optimise health services to improve access to medicines in a particular kind of setting.

### Medicines: from availability to access

In India, as in many other LMICs, various issues hinder access to medicines. The access issue is more acute for NCD patients due to their need for lifelong supply. Issues related to procurement, management of supply chain and the governance of drugs procurement systems in the state level are crucial to ensuring availability of medicines at PHCs. Since drugs procurement and supply is often relatively centralised in many states, those states that have implemented reforms of their procurement systems are better-off with respect to ensuring availability of medicines in PHCs [46]. However, in addition to reforms at the state level, district and sub-district level capacity and performance issues are crucial to strong health systems. In most Indian states, local health systems are increasingly being strengthened through decentralisation reforms and understanding how such local initiatives at district and sub-district levels work is crucial to improving responsiveness of health systems to emerging challenges of providing good quality NCD care.

Access to medicines is much more than ensuring availability. In spite of free medicines and care at PHCs on paper, various reasons related to availability of services, acceptability of care, health-seeking behaviour of patients and perceived and technical quality of medicines and healthcare at government PHCs hinder utilisation of medicines, even where medicines are available. In the present study, we hope to better understand how this situation could be changed in favour of improving access to medicines.

### NCD in primary health care

Our intervention somewhat mirrors the much-criticized vertical disease-control approach as it focuses on improving access to medicines for NCD patients *specifically* (and thus not for patients presenting with other problems) and on strengthening processes at the primary level for the care of NCD patients *specifically* (and, again, not for patients presenting with other problems), while not addressing the various underlying reasons for the *general* under-performance of local health systems. A more integrated and less selective approach would have been more appropriate in addressing PHC-wide issues in drugs management and continuity of care rather than take up these issues only in relation to NCDs. The intervention by highlighting NCDs may in fact in some areas decrease focus on other services or activities that are less in the picture and therefore less well-resourced. To some extent, this may be addressed by ensuring that the intervention warrants that health workers privilege their existing services whenever there is a need to choose one against the other. In terms of sustainability of the intervention, since the most of the government health services in India do not currently provide systematic care and follow-up for NCDs, we foresee that the generation of research evidence and positive experiences by the district team during the ATM intervention may result in the incorporation of some of its intervention activities into their annual action plans.

## Additional files

Additional file 1: Rapid local health system assessment. This assessment of health system performance made use of an expanded version of WHO health system framework by Van Olmen et al. (PDF 145 kb) Additional file 2: Random.org. The screenshot of randomisation of PHCs using open source website RANDOM.ORG. (JPG 55 kb) Additional file 3: Intervention plan document for the ATM study. This document provides a step by step plan for the interventions of the study, along with the rationale. (PDF 1098 kb) Additional file 4: Household survey tool. Household survey tool used in baseline survey of the ATM study. (PDF 159 kb) Additional file 5: Facility survey tool. Facility survey tool used in baseline survey of the ATM study. (PDF 318 kb) Additional file 6: Exit interview tool. Exit interview tool used in baseline survey of the ATM study. (PDF 150 kb) Additional file 7: Information consent. Information sheet used for obtaining consent in household survey of the ATM study. (PDF 428 kb) Additional file 8: Supervisor structured checklist for household survey. Checklist for the supervisor to validate on field data collection in the household survey of the ATM study. (PDF 75 kb) Additional file 9: Drug quality tests. This document provides rationale and step by step narration for drug quality testing activities conducted under ATM study. (PDF 54 kb)

## Abbreviations

ANM: Auxiliary nurse-midwife
ARS: Arogya Raksha Samiti (Health protection committees)
ASHA: Accredited social health activist
ATM: Access to medicines
BCC: Behavioural change communication
FGD: Focus group discussion
LMIC: Low- and middle-income country
LSCS: Lower segment caesarean section
MO: Medical officer
NCD: Non-communicable diseases
NHM: National Health Mission
OOP: Out-of-pocket expenditure
PHC: Primary health centre
WHO: World Health Organization
