# Supplementary material for: Improving access to medicines for non-communicable diseases in rural India: a mixed methods study protocol using quasi-experimental design
Source: BMC Health Serv Res. 2016 Aug 22;16(1):421. doi: 10.1186/s12913-016-1680-3 (PMC4994301; doi:10.1186/s12913-016-1680-3)
Supplement: Additional file 1: — Rapid local health system assessment. This assessment of health system performance made use of an expanded version of WHO health system framework by Van Olmen et al. (PDF 145 kb) [file 12913_2016_1680_MOESM1_ESM.pdf]

Annex 1: Rapid local health system assessment

The authors expanded the building blocks' framework of the WHO health systems framework, and included four new elements — population, context, goals and values — while visualising the dynamic relationships and reciprocal interactions between the elements. The *'health system dynamics'* framework states that all ten elements are not equal: it emphasises a central axis between governance, human resources, service delivery and population. While dealing with health system performance, it views health systems explicitly as social systems that are embedded in a context that shapes its design and development and that in turn emanate the prevailing values of the society to which they belong.

This vision implies a central role for the population, on the receiving end as patients and, via representation and other means, as citizens in governance of the health system. In addition, it emphasizes that choices made in steering of health systems are *de facto* based upon a context-specific balance in values and principles, and thus by power relations between stakeholders (2Van Olmen et. al. BMC Public Health, 2012).

We incorporated the elements of this framework in designing a rapid assessment tool, with a bias towards the possible health system components that are a basic minimum for implementing healthcare for NCDs. We hence developed a tool that consists of proxy indicators (or assessment criteria wherever quantitative indicators were not possible or feasible) of particular functions within each of these health system components. In choosing these functions, we have borne in mind that these indicators should reflect a critical function under a particular component that is a basic minimum for organising and providing care for NCDs. For example, a stable leadership/governance as indicated by a stable and qualified health manager at the taluka level (a Taluka Health Officer) is a basic minimum in terms of governance for ensuring functional network of PHCs in that taluka (see Table 1).

The 10 *talukas* vary widely in terms of socio-economic development indicators. (Table 2) The state government-appointed committee for addressing regional disparities in development categorized *talukas* across the state based on various health, literacy, socio-economic, political, and economic indices. In Tumkur, only the headquarter taluka (Tumkur) was categorised as relatively developed. The other nine were classified as being "backward" to "most backward". Considering that the intervention is dependent on several health system factors for it to succeed, it was necessary to assess if all (or how many) of the 10 *talukas* have the necessary conditions to implement the proposed interventions (health services and community mechanisms strengthening to improve access to medicines for non-communicable diseases). Hence we devised a tool for this assessment using the health system framework by Van Olmen et al (figure 1).

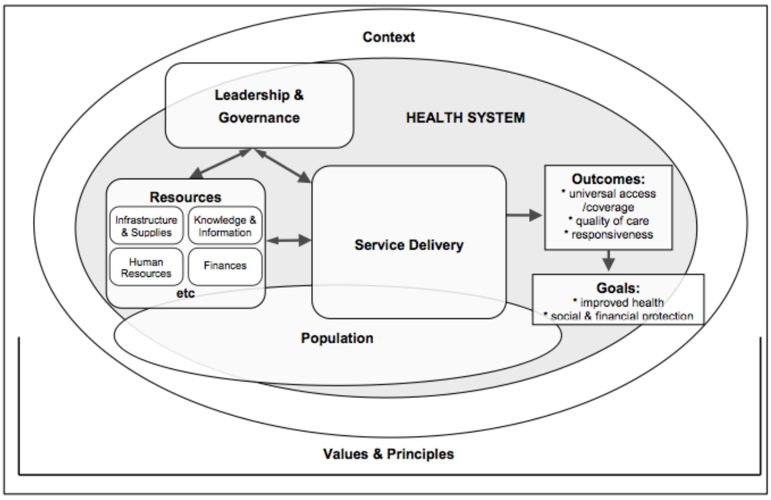

Figure 1: Health system framework by Olmen et al.
Image source: van Olmen, J., Marchal, B., Damme, W. Van, Kegels, G., & Hill, P. S. (2012). Health systems frameworks in their political context: framing divergent agendas. *BMC Public Health*. http://doi.org/10.1186/1471-2458-12-774

We devised indicators for the various components of the taluka health system(*table 4*). Based on this assessment, the talukas where the necessary health system conditions for implementing the intervention did not exist were excluded (three *talukas* were excluded). We chose three *talukas* randomly from among the remaining seven using random number generating calculator (Available at www.random.org). The *talukas* are Koratagere, Sira and Turuvekere (as shown in figure 1). In these three *talukas*, PHCs will be randomly allocated to one of the three arms of the intervention (A, B and C). A brief socio-demographic profile, health and development indicators of the three talukas are shown in *table 3*. The intervention packages will be implemented in A and B, while C will be the control.

Table 2: Tumkur talukas categorised by socio-political-economic development

**Category**	**Taluka**
Relatively Developed Taluks	Tumkur
Backward Taluks	Tiptur Koratagere
More Backward Taluks	Turuvekere Madhugiri Chikkanayakana Halli
Most Backward Taluks	Sira Pavagada Gubbi Kunigal

(Source: Based on Karnataka government high-powered committee on addressing regional imbalances)

Table 4 : Taluka health system assessment tool.

Component	What to assess?	How to assess? Indicator/assessment criterion	Justification
Leadership and Governance	Stable leadership at taluka level	Permanent THO<br>Tenure of present THO (since when is he in this post)	Presence of a qualified THO (as opposed to temporary in- charge THOs) is crucial for taluka leadership and supervision
	Functioning community participation platforms (Village health & sanitation committee) and Arogya Raksha Samiti (patient welfare committee at PHCs)	Proportion of "functional" VHSCs in taluka (expressed as %age)<br>Proportion of "functional" ARS (3tilized3 as %age)<br>Proportion of ARS committees holding regular meetings (>3 meetings last year)<br>Qualitative assessment of constructive engagement by ARS & community	Their functioning is crucial for one of the hypothesis that is being tested: 3tilized3 group of NCD patients could "push" improved access using existing community participation platforms
Infrastructure	Private healthcare infrastructure	Number of private nursing homes in taluka<br>Number of private clinics (single-doctor outpatient clinics)<br>Number of private pharmacies	Private sector dependence in health especially for OP care is high in India.
	Government infractructure	% PHCs with own building % sub-centres with own building % PHCs with a designated pharmacy % PHCs with a designated laboratory	PHCs with designated pharmacy and laboratory are necessary for NCD care
Human Resource	Doctors	% PHC with at least 1 qualified (MBBS) doctor % of doctor posts filled<br>% of permanent doctors (as opposed to contracted-in/temporary/AYUSH)	
	Pharmacists	% PHCs with a full-time working pharmacist	
	Laboratory technicians	% PHCs with a full-time working laboratory technician	
	Other health staff	% vacancy of ANMs % vacancy of ASHAs Average population/ASHA	Auxiliary nurse- midwives (ANM) and accredited social health activists (ASHA) play crucial role
			in outreach, referral, preventive care and follow-up
Service delivery	Utilisation and coverage of services	Average OP 4 utilised4on rate % institutional delivery TB case detection rate TB cure rate	Crude indicator of 4 utilised4on of PHC services.<br>Tuberculosis being a comparable chronic condition requiring community follow-up

			and repeated visits
Financing	Utilisation of available funds		% untied funds utilised till date this year
Health status	Mortality rate	Infant mortality rate	A crude indicator of health status of the population that is related to health services performance
