# Supplementary figures and images for: Improving access to medicines for non-communicable diseases in rural India: a mixed methods study protocol using quasi-experimental design
Source: BMC Health Serv Res. 2016 Aug 22;16(1):421. doi: 10.1186/s12913-016-1680-3 (PMC4994301; doi:10.1186/s12913-016-1680-3)

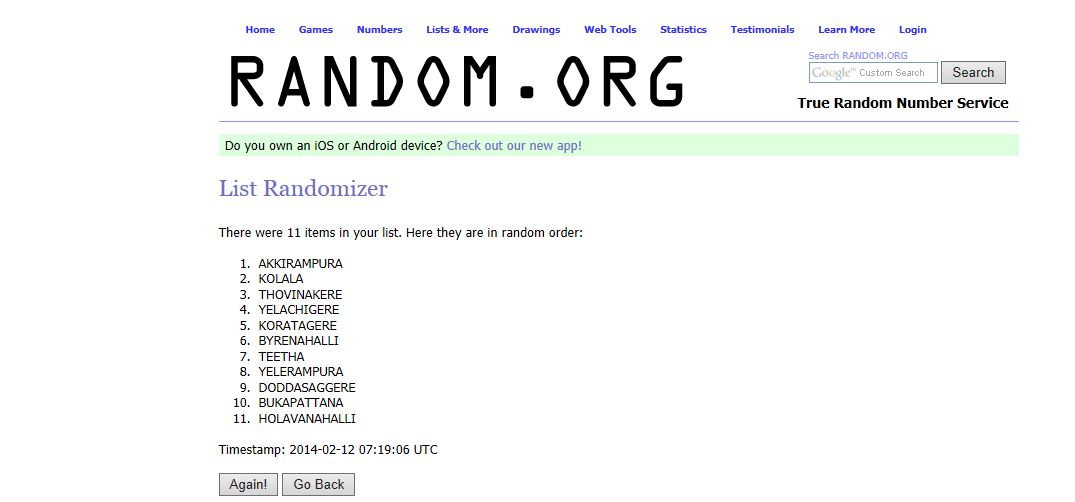

Supplement: Additional file 2: — Random.org. The screenshot of randomisation of PHCs using open source website RANDOM.ORG. (JPG 55 kb) [file 12913_2016_1680_MOESM2_ESM.jpg]
