# Supplementary material for: Improving access to medicines for non-communicable diseases in rural India: a mixed methods study protocol using quasi-experimental design
Source: BMC Health Serv Res. 2016 Aug 22;16(1):421. doi: 10.1186/s12913-016-1680-3 (PMC4994301; doi:10.1186/s12913-016-1680-3)
Supplement: Additional file 3: — Intervention plan document for the ATM study. This document provides a step by step plan for the interventions of the study, along with the rationale. (PDF 1098 kb) [file 12913_2016_1680_MOESM3_ESM.pdf]

Equitable access to quality generic medicines for patients with NCD in Tumkur, India: A health systems research

Intervention plan document

Maya Annie Elias, Manoj K Pati, Praveen Aivalli, Bhanuprakash, Mune Gowda, and NS Prashanth

(with inputs from Bart Criel, , N Devadasan & Sadhana SM)

Introduction

Institute of Public Health, Bangalore (IPH), Karnataka State Health Systems Resource Centre, Bangalore (KSHSRC) and Institute of Tropical Medicine, Antwerp (ITM) are jointly conducting a research study titled "Improving equitable access to quality generic medicines for patients with Non Communicable Diseases" in Tumkur district, Karnataka. The study is being supported by WHO-Alliance for Health Policy & Systems Research (WHO-Alliance) for a period of three years from May 2013- April 2016.

This study seeks to understand if (and how) improvements in health services and strengthening of community mechanisms could improve access to medicines for people with non-communicable diseases (diabetes and hypertension) in Tumkur district. It is designed as a *quasi-experimental study using a cluster-randomized control trial design*. An intervention at taluka-level will be implemented and its effects will be studied through a baseline-endline survey among households and health facilities. A mixed methods approach will be used to verify and refine the initial programme theory of the intervention and understand the PHC-level changes in response to such an intervention.

We first began taluka selection with a taluka health systems assessment, in which we excluded three talukas as not meeting the criteria for implementation of the intervention. From the other seven talukas, three talukas were chosen randomly. The talukas are Koratagere, Turuvekere and Sira. PHCs in these talukas will be randomly allocated to three arms of the intervention: community platforms strengthening (A), community platforms strengthening + health services optimisation (AB) and control (C). The baseline and endline survey make use of standardized household and facility survey tools that were developed and tested in low- and middle-income country (LMIC) settings by WHO. We will use qualitative methods (in-depth interviews, focus group discussions and observation notes of field visits) during and after the intervention to understand *how* health workers and patients within the PHCs, taluka and district level responded to the intervention.

The purpose of this document is:

1. To provide a step by step plan for the proposed intervention of the study, along with the rationale
2. To aid as a reference manual for the research team throughout the project intervention period

Background

India is going through major transition with regards to its socioeconomic parameters, demographic profile and disease patterns. Along with the high burden of communicable diseases, non-communicable diseases are also on the rise. We are supposed to be the diabetic capital of the world with the highest number of diabetic and hypertensive patients[1]. Non-communicable diseases contributed to two-thirds of all deaths globally in 2011 and around 80% of such NCD deaths occurred in low- and middle-income countries[2]. According to the WHO estimates, non-communicable diseases in India accounted for an estimated 53% of all mortality in 2011[3]. While disability-adjusted life-years (DALY) due to NCDs and associated economic implications have become topics of interest for public health researchers, other important aspects of greater interest to policymakers is the cost of treatment and the ways to improve access to essential medicines. Inadequate resource allocation, supply and management of medicines in the public sector and resultant high out-of-pocket (OOP) expediture have been highlighed as important issues in NCD care[4].

Globally, there has been a lot of discussion on organising care for chronic diseases, especially in LMICs. The 2012 United Nations General Assembly declaration on chronic diseases was a major step in this direction. It emphasized the need for coordinated action among all nations to deal with the NCD burden and put forth various intervention strategies such as improving access to medicines, better technology and international collaborative efforts for NCD control[5].

In all the debates related to NCDs, it has been pointed out that a continuous, integrated healthcare delivery system is a prerequisite for the required care for NCDs. But often we see that the health information systems for NCDs are weak in most LMICs and healthcare delivery systems in these countries are more tuned

---

[1]Patel, V., Chatterji, S., Chisholm, D., Ebrahim, S., Gopalakrishna, G., Mathers, C., ... Reddy, K. S. (2011). Chronic diseases and injuries in India. *Lancet*, *377*(9763), 413–28. doi:10.1016/S0140-6736(10)61188-9

[2] WHO | Noncommunicable diseases. (n.d.). Retrieved from http://www.who.int/mediacentre/factsheets/fs355/en/

[3]World Health Organization. (2011c). WHO NCD Country Profiles. Geneva: World Health Organization.

[4] Bhojani, U., Thriveni, B., Devadasan, R., Munegowda, C., Devadasan, N., Kolsteren, P., & Criel, B. (2012). Out-of-pocket healthcare payments on chronic conditions impoverish urban poor in Bangalore, India. *BMC public health*, *12*(1), 990. doi:10.1186/1471-2458-12-990

[5]United Nations General Assembly. Resolution adopted by the General Assembly: 66/2: Political Declaration of the High-level Meeting of the General Assembly on the Prevention and Control of Non-communicable Diseases. Adopted September 19, 2011; published January 24, 2012.

to deal with the acute and infectious diseases rather than chronic conditions[6]. A recent review of evidence on burden of chronic diseases and possible responses from the health system highlights, among others "strengthened public health and primary healthcare systems"[7]. Good primary health care, supported by family and self-care is believed to be the backbone of cost-effective NCD care[8].

Community and health services intervention models have been tried out to improve the care for NCDs in both high-income and LMIC settings. Community interventions for NCDs mainly concentrate on prevention and many countries have successfully implemented awareness generation and lifestyle modification programmes[9]. Health services interventions primarily focus on improving compliance (through better follow-up, use of telephones or through health workers) and improving the information basis for lifestyle modification advice by health workers (building capacities of health workers to deliver lifestyle modification advice, nurse- or pharmacist-aided counseling)[10,11].

Another intervention suggested for effective NCD control is community involvement in planning, implementation and monitoring of NCD management programmes and advocacy for increasing the governments' commitment towards NCDs[12]. In India, there is a long history of such civil society involvement

---

[6]Dans, A., Ng, N., Varghese, C., Tai, E. S., Firestone, R., & Bonita, R. (2011). The rise of chronic non-communicable diseases in southeast Asia: time for action. *Lancet*, *377*(9766), 680–9. doi:10.1016/S0140-6736(10)61506-1

[7]Patel, V., Chatterji, S., Chisholm, D., Ebrahim, S., Gopalakrishna, G., Mathers, C., … Reddy, K. S. (2011). Chronic diseases and injuries in India. *Lancet*, *377*(9763), 413–28. doi:10.1016/S0140-6736(10)61188-9

[8]Beaglehole R, Epping-Jordan J, Patel V, Chopra M, Ebrahim S, et al. (2008) Improving the prevention and management of chronic disease in low-income and middle-income countries: a priority for primary health care. Lancet 372: 940–994.doi: 10.1016/S0140-6736(08)61404-X

[9]Puoane, T. R., Tsolekile, L., & Sanders, D.(2013). A case study of community-level intervention for non-communicable diseases in khayelitsha , Cape Town Empowerment of Women and Girls. Institute of Development Studies , University of the Western Cape.

[10]Hirimuthugoda, L. K., Wathudura, S. P. K., Edirimanna, H., Vithanage, T. K., & de Silva, P. A. (2013). Experimental design: impact of an intervention to improve clinic attendance of patients with non-communicable diseases through telephone follow-up. *The Lancet*, *381*, S63. doi:10.1016/S0140-6736(13)61317-3

[11]Saleem, F., Hassali, M. a, Shafie, A. a, Ul Haq, N., Farooqui, M., Aljadhay, H., & Ahmad, F. U. D. (2013). Pharmacist intervention in improving hypertension-related knowledge, treatment medication adherence and health-related quality of life: a non-clinical randomized controlled trial. *Health expectations : an international journal of public participation in health care and health policy*. doi:10.1111/hex.12101

[12]Bonita, R., Magnusson, R., Bovet, P., Zhao, D., Malta, D. C., Geneau, R., Beaglehole, R. (2013). Country actions to meet UN commitments on non-communicable diseases: a stepwise approach. *Lancet*, *381*(9866), 575–84. doi:10.1016/S0140-6736(12)61993-

in healthcare and the National Rural Health Mission (NRHM)[13] has formalized the channel for this engagement through the formation of Village Health and Sanitation Committees (VHSC) at the village level and *Arogya Raksha Samithis* (ARS; health protection committees in the local language, Kannada) at the PHC level. Similar committees have been formed at secondary and tertiary hospital levels. Though ARS committees are expected to play an active role in supporting and improving the overall quality of services, studies conducted across different states report that these committees often lack the understanding of their expected role and suggest capacity-building as a means to improve their functioning[14,15].

It is in this context that we propose the interventions under the ATM project, for strengthening the community participation platforms and optimising the health services to improve access to medicines for NCDs in Tumkur.

The details about data collection, the tools used, analysis proposed and dissemination of lessons learnt are available in the study protocol. The study protocol has received approval from the ethics committees of WHO, Geneva and IPH, Bangalore. The Government of Karnataka has provided permission to conduct the study in Tumkur.

Intervention plan

Study setting

Tumkur district is one of the 30 districts in Karnataka state in southern India with a population of 2.67 million (2011 census). There is a mix of government and private sector, formal and informal providers as well as a range of single doctor clinics to secondary and tertiary level hospitals. Despite significant achievements in providing and managing health services, inter-district disparities in health outcomes persist. A recent government task force categorized Tumkur as an average district with respect to health and development outcomes. Tumkur, in terms of performance of health services and health outcomes, is comparable to most other districts in Karnataka. IPH has

[13] The National Rural Health Mission was launched in 2005. Under the NRHM, the Indian government committed itself to increasing its expenditure on health (then estimated to be less than 0.9% of its GDP). NRHM listed "communitization" of the health services as one of its core strategies for improving the quality and performance of the Indian health system

[14]An exploratory study of VHSC and ARS functioning. (2012). Karnataka State Health System Resource Centre, Bangalore

[15]Adsul, N., & Kar, M. (2013). Study of rogi kalyan samitis in strengthening health systems under national rural health mission, district pune, maharashtra. *Indian journal of community medicine : official publication of Indian Association of Preventive & Social Medicine*, *38*(4), 223–8. doi:10.4103/0970-0218.120157

been working with the Tumkur district health team since 2009. IPH's focus in Tumkur has been to strengthen the management capacities of the Tumkur district and taluka teams as well as conducting operational research, problem-solving visits and action research at PHC level. In figure 2, the talukas (administrative sub-divisions of districts) of Tumkur are shown highlighting the talukas chosen for the ATM study.

Selection of study *talukas*- Rapid health system assessment

Tumkur has 10 *talukas*[16] and they vary widely in terms of socio-economic development indicators. The state government-appointed committee for addressing regional disparities in development categorized *talukas* across the state based on various health, literacy, socio-economic, political, and economic indices. In Tumkur, only the headquarter taluka (Tumkur) was categorised as relatively developed. The other nine were classified as being "backward" to "most backward". Considering that the intervention is dependent on several health system factors for it to succeed, it was necessary to assess if all (or how many) of the 10 *talukas* have the necessary conditions to implement the proposed interventions (health services and community mechanisms strengthening to improve access to medicines for non-communicable diseases). Hence we devised a tool for this assessment using the health system framework by Van Olmen et al (figure 1)[17].

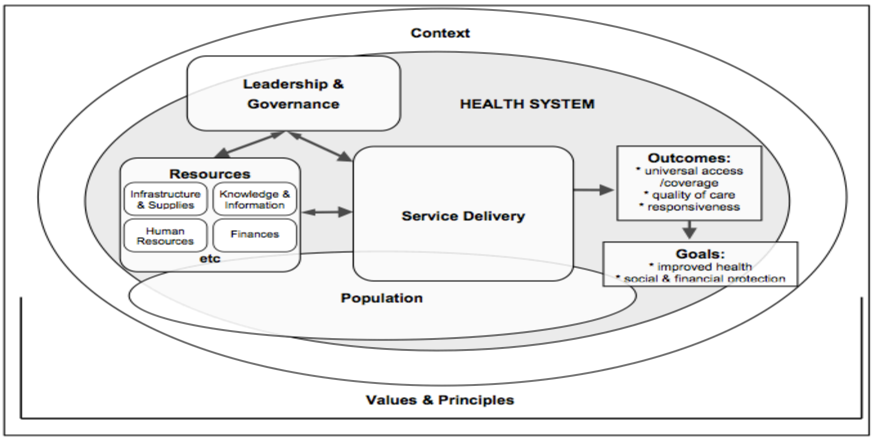

Figure 1: Health system framework by Olmen et al

Image source: Image source: van Olmen, J., Marchal, B., Damme, W. Van, Kegels, G., & Hill, P. S. (2012). Health systems frameworks in their political context: framing divergent agendas. *BMC Public Health*. http://doi.org/10.1186/1471-2458-12-774

[16] A *Taluka* is an administrative sub-division of district. In Karnataka, a typical taluka has a population of a few hundred thousand people. *Panchayats (*local governments with elected representatives) administer the health, education and social welfare services at the district, taluka and village level.

[17]van Olmen, J., Marchal, B., Damme, W. Van, Kegels, G., & Hill, P. S. (2012). Health systems frameworks in their political context: framing divergent agendas. *BMC Public Health*. http://doi.org/10.1186/1471-2458-12-774

We devised indicators for the various components of the taluka health system and based on this assessment, the talukas where the necessary health system conditions for implementing the intervention did not exist were dropped (three *talukas* were dropped). We chose three *talukas* randomly from among the remaining seven. The *talukas* are Koratagere, Sira and Turuvekere (as shown in figure 1). In these three *talukas*, PHCs will be randomly allocated to one of the three arms of the intervention (A, B and C). A brief socio-demographic profile, health and development indicators of the three talukas are shown in *table 1*. The intervention packages will be implemented in A and B, while C will be the control. A will receive Package A and B will receive both types of intervention packages.

Table 1: Health and development indicators of the study talukas

**Indicators**	**Kortagere**	**Sira**	**Turvekere**
Area	652Sq km	1552Sqkm	778Sqkm
Population	160952	301473	174297
Number of private pharmacies	19	30	22
General literacy rate	71%	67%	73%
Number of PHCs	11	17	11
Avg. Population per PHC,	14500	18000	16000
Number of private hospitals/clinics	21	64	18
IMR	12	10	3
Number of PHCs with qualified MBBS doctors	11	15	10

Package A: Strengthening community participation platforms

In these PHCs, the intervention will focus on the existing community participation platforms. The objective is to strengthen these platforms to become pressure groups for better NCD care in the PHCs. Two third of the PHCs from the selected *talukas* will receive this package.

The two key assumptions underlying this package is the following:

1. Health workers of PHCs can themselves encourage and empower ARS[18] and VHSC[19] members to engage with the PHC team to improve access to

---

[18] The Arogya Raksha Samiti (ARS; Health protection committee) are constituted at the health facility level with a mix of health workers, elected representatives of the local government and other community members. Under NRHM, ARS committees have been vested with important oversight responsibilities and financing arrangements.

[19] Village health and sanitation committees (VHSC) is another body set up under the NRHM at each of the villages. The ASHA is a key member of the VHSC. VHSCs are

medicines for NCD through provision of information about NCDs, NCD care and entitlements to such care at local PHCs

2. Existing community participation platforms can trigger improvements in PHCs if their members are made aware of their roles and responsibilities and if their engagement is facilitated by health workers

In the community package, workshops will be held at each of the PHCs with the PHC health workers to help them facilitate the participation of ARS and VHSC members in discussions about NCD care at their PHCs, need for and availability of medicines for NCD at local PHCs and about lifestyle modification for secondary and primary prevention of NCDs. We will also seek to place NCD as a topic for public discussion during the one or more PHC health and nutrition days that are routinely conducted at the PHC, where several community members participate. At present, these events are largely focusing on reproductive and child health services.

The following diagram explains the intervention logic. The choice of intervention inputs and the likely processes through which the expected output may be seen are based on existing theory on what works (body of literature) and our assumptions (based on our understanding of the study setting).

---

expected to function as a key link between health worker responsibilities and community expectations at the village level. They also have minimal financial allocation.

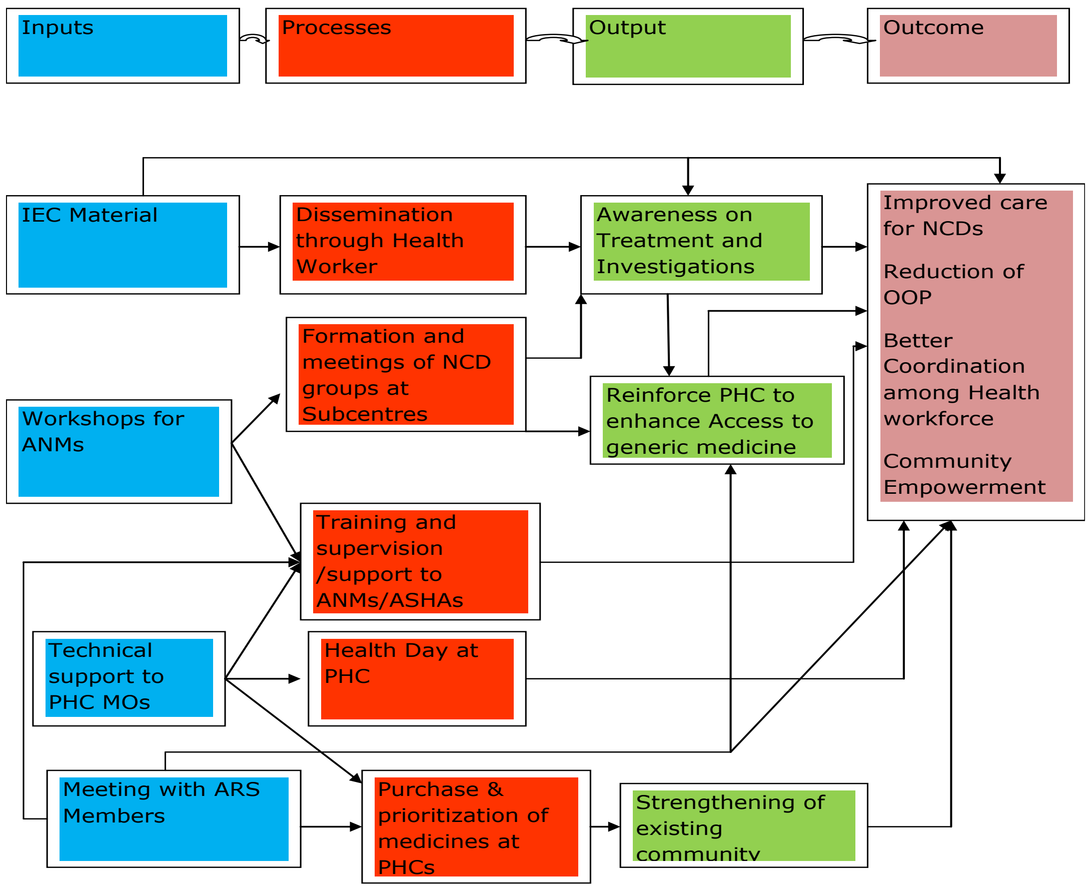

Figure 3: Intervention logic of package A.

Table 2: Inputs and activities planned under Package A

Inputs	Activity	Expected outcome
**Source/finalise awareness material**	Compile awareness material (pamphlets, posters, street play themes and wall-painting templates) in Kannada (local language) from all sources.<br><br>Awareness material shall focus on (1) lifestyle modification for NCD, (2) need for long-term follow-up and medication for NCD, and (3) availability of free generic medicines for NCD at local PHCs<br><br>Obtain feedback from health workers on their utility for awareness activities at PHC and village level<br><br>Disseminate material among health workers, ARS and VHSCs	Improved awareness among people about NCD treatment and primary/secondary prevention<br><br>Increased utilization of PHCs for treatment for NCD<br><br>Increased pressure on PHC for stocking and providing medicines for NCD
**Workshops for PHC health workers (ANMs, Anganwadi workers and**	Orientation to health workers on:<br>(1) lifestyle modification for NCD, (2) need for long-term follow-up and medication for NCD, and (3) availability of free generic medicines for NCD at local PHCs	Improved health worker knowledge on NCDs<br><br>Discussion on NCDs in ARS and VHSCs<br><br>Formation of NCD patient groups

**ASHAs[20])**	How to impart information about diabetes and hypertension to community members using the awareness material<br><br>How to organize patients and form patient groups<br><br>Involving ARS and VHSC members in improving utilization of PHC services	Improved coverage of patients with NCD as measured by increased proportion of NCD patients within the PHC's designated population, who seek and receive regular treatment for NCD at selected PHCs
**Formation of NCD patient groups**	Support to the ANMs in selected PHCs to organise NCD patients and inform about the importance of regular treatment and the advantages of generic medicines<br><br>NCD patient groups work with the ARS and VHCs to ensure availability of generic drugs for NCDs at PHC.	Better awareness in the community leading to increased registration of new patients<br><br>Decrease in stock-out of NCD drugs at PHC
**IEC at community level**	The health workers of the intervention PHCs will receive a folder containing<br>- Information leaflets about diabetes & hypertension<br>- Information about the designated NCD check up day<br>- The PHC health day event held 1-3 times per year at the PHC will also include activities related to NCD, led by ARS members and health workers	Better awareness among community members about the illness and treatment<br><br>Increased patient registration at PHCS<br><br>Better treatment adherence<br>Decreased out-of-pocket expenditure

[20] Auxiliary Nurse-midwife (ANM) is the health worker in charge of a sub-centre and is a trained nurse-midwife with a large focus on reproductive and child health and other disease-control programmes. Anganwadi workers are in charge of pre-schools at village level. ASHAs are a cadre of community health workers, established under the NRHM in each village; a local woman volunteer trained formally to improve access and utilization of services, presently with a large focus on reproductive and child health.

	Also the information about diabetes and hypertension will be displayed at prominent places in the PHC and local villages	
**Meeting with ARS members**	Orientation/ capacity building for ARS members about their functions and possibility of utilizing untied funds for purchasing medicines<br><br>Facilitate their interaction with patient groups and PHC staff	Decrease in stock-outs of NCD drugs at PHC due to ARS involvement and use of untied funds<br><br>Better coordination between ARS members and community

Package B: Health service optimization
One-third of the PHCs in the study talukas would receive inputs for optimizing their health services so that NCD patients could be registered at their PHC and periodic follow-up could be started. At present, PHCs are not geared to provide continuous care for people with NCD. For example, most PHCs in Tumkur do not use patient or family cards. The use of patient or family cards is vital to ensure continuous care for people with NCD. In addition, the use of simple clinical protocols for diagnosis and management of diabetes and hypertension at primary health care are available, but rarely followed.

The logic of Package B is that merely the formation of patient groups and pressure on existing community participation platforms will be inadequate to improve the provision of good quality NCD care at the PHC. The community level activities will increase the number of patients seeking care from PHC, whereas there is a need to ensure that the PHC is able to provide good quality care for NCDs. PHCs that receive Package B PHCs shall also receive inputs to optimize their existing outpatient consultation arrangements to accommodate the specific needs of ensuring continuous care for people with NCD (sufficient consultation time to allow for counseling on lifestyle issues, medicines as per established principles of rational treatment and regular follow-up through patient-held case records).

The intervention assumptions could be summarized as follows:

(1) PHCs in Tumkur are focused on care for acute episodes of infectious diseases; they require inputs to help modify their outpatient care process for ensuring continuous care for NCD
(2) Improving access to medicines for NCD at PHCs requires support to improve service-delivery arrangements at the PHC, including a system of registration of patients, implementation of patient-held treatment records, increased consultation time for counseling on lifestyle modification and involvement of health workers (especially pharmacists).
(3) Community-level interventions to improve access to medicines should be accompanied by health services interventions to improve availability and care for NCD in order to improve access to medicines for NCD.

The proposed intervention logic is depicted in *figure 4*. Health workers will not respond uniformly to these inputs. Moreover, several local relationships and dynamics within the PHCs are likely to affect (and be affected by) the intervention inputs. However, we hope to be able to understand which community platforms and/or PHCs respond positively and how their response affects (or does not?) the NCD care and access to medicines for NCD at the PHC.

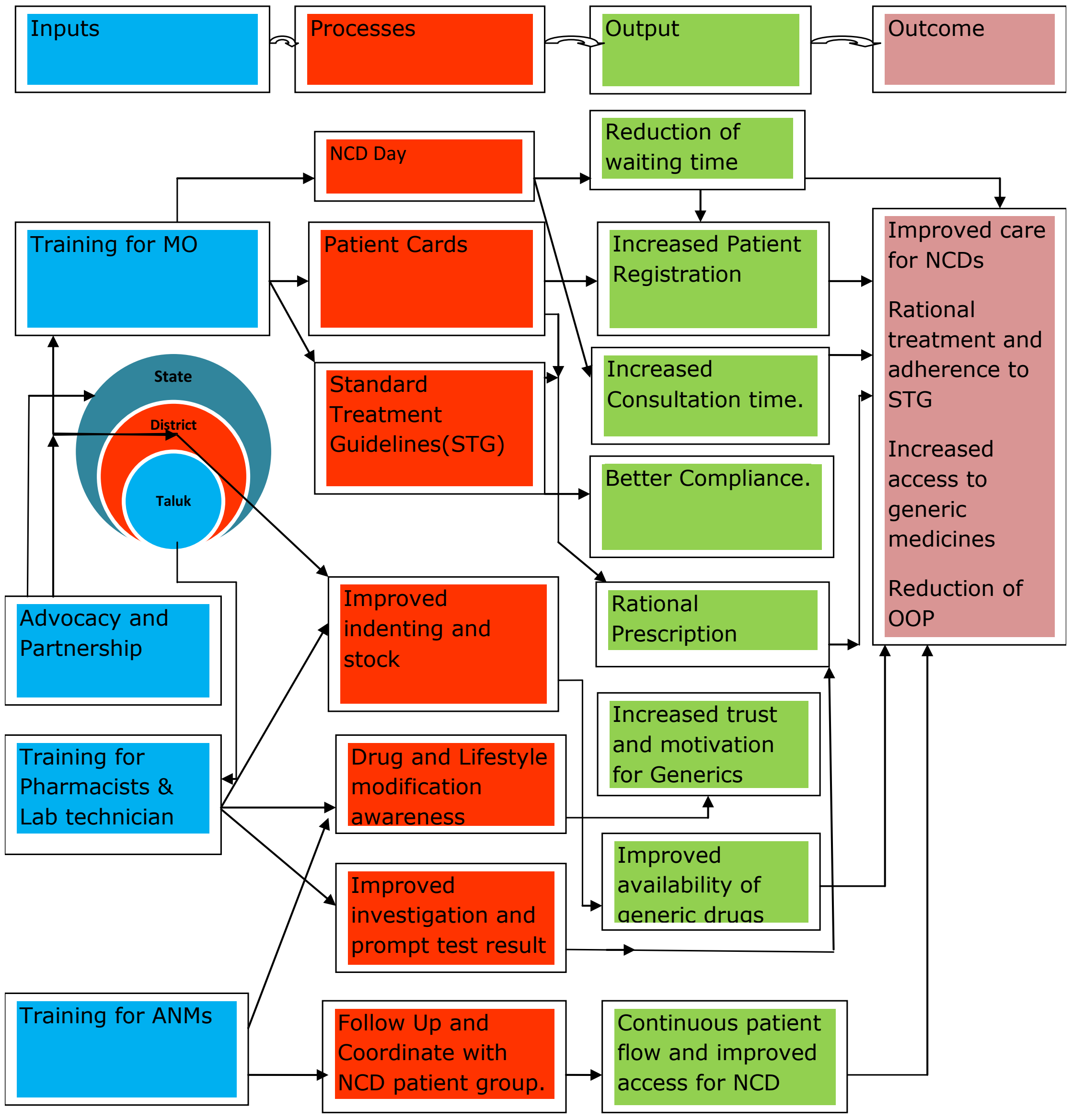

Figure 4: Intervention logic of Package B

Table 3: Inputs and activities planned under Package B (Package A is also included in this package. See table 1 for Package A)

Inputs	Activity package	Expected outcome
**Orientation training & workshop for PHC medical officers**	Orientation to MOs in the selected PHCs on<br>- Standard treatment guidelines for NCDs<br>- Rational prescription<br>- Maintaining treatment cards for patients with NCDs<br>During the training the need for increased consultation time for patients will be stressed and they will be encouraged to include NCD management as an agenda for community health days of the PHC	- Improved awareness on treatment protocol<br>- Rational prescription & focus on lifestyle modification<br>-
**Workshop for pharmacists**	Training will focus on<br>Need assessment for medicines<br>Indenting for medicines<br>Record keeping at PHCs<br>Role of pharmacists in providing advice to patients on medicines, side-effects and counselling on non-drug treatment for NCD. Pharmacists will be encouraged to give provide these inputs to patients while disbursing the medicines at PHCs	- Better medicine availability- reduced stock outs<br>- Improved patient awareness on medicines & lifestyle modification<br>- Better record-keeping system in PHCs
**Workshop for ANMs, ASHAs and Anganwadi workers**	In addition to activities under Package A, in these PHCs, the focus would be to help ANMs and ASHAs with follow-up of NCD patients in their area and ensuring that they visit the PHC regularly for follow-up and medicines.	- Better patient awareness on NCDs resulting in better patient flow<br>- Better coordination between health workers and community
**Advocacy and coordination**	Coordination and advocacy at state, district and taluka levels with different stakeholders to ensure supply of drugs to the PHCs which ask for these drugs either through routine supply chain or through district health action plans (as an innovation) or through procurement from local funds (ARS)	- Improved medicine supply<br>- Reduced stock outs<br>- Improved quality of medicines

**Registration of NCD patients and follow-up using patient-retained medical records**	Pharmacists will ensure implementation of:<br>- Registration and issue of patient-retained medical records to all patients with Diabetes and Hypertension that come for outpatient consultation at the PHC<br>- Monthly follow-up dates will be given to all patients. PHCs will be encouraged to designate a monthly NCD clinic day to ensure that sufficient consultation time may be given<br>- Pharmacists participate in providing lifestyle modification advice as well as clear and detailed instructions to all patients on how to take medication (unlike short-term episodic medicines for infectious diseases)	- Increased patient registration<br>- Improved treatment compliance of NCD patients due to patient-retained records Improved utilization of PHC services for NCD<br>- Improved retention of patietns at PHC , improved compliance and follow-up

Access to Medicine Activites till August 2015 - GANTT Chart

Activities	Target	Subactivities	Persons Responsible	Feb-14			Mar-14				Apr-14					May-14					Jun-14			
				WK2	WK3	WK4	WK1	WK2	WK3	WK4	WK1	WK2	WK3	WK4	Wk5	WK1	WK2	WK3	WK4	Wk5	WK1	WK2	WK3	WK4
Finalise awareness materials for community and patient awareness		Literature Search- Awareness Material	Manoj, Prashanth				■	■	■															
		Compile and organise the awareness material	Maya, Prashanth						■	■														
	Enough pamplets to distribute-50000 Nos Initially	Preparation of Pamphlets-Content & Designing	Maya,Manoj					■	■	■														
		Production of Pamphlets- Printing and Procurement	Praveen,Muna,Bhanu								■													
	Enough posters for all primary facilities- PHCs, SCs, GP offices-400 Nos	Preparation of Posters-Content & Designing	Maya,Manoj					■	■	■														
		Production of Posters- Printing and Procurement	Praveen,Muna,Bhanu								■													
	At least 100 health cards per PHC	Preparation of Medical Record Book (Health Card)-Content & Designing	Maya,Manoj, Mos					■	■	■														
		Production of Medical Record Book (Health Card)- Printing and Procurement	Praveen,Muna,Bhanu								■													
	1-2 Wall paintings per PHC at strategic places	Preparation of Wall Paintings-Content & Designing	Maya, Manoj, Prashanth,MO					■	■	■														
		Production of Wall Paintings- Identifying and Hiring artist (one/ Taluk)	Manoj,Praveen,Muna, Bhanu						■	■														
		Display of Wall Paintings- Painting at strategic places	MO, Praveen,Bhanu,Muna								■	■	■	■	■									
	2-3 Street Theatre per PHC Per Year	Identifying Street Theatre Group	Prashanth, Manoj, Bhanu								■	■												
		Hiring a Street Theatre Group	Prashanth, Manoj, Bhanu										■											
		Preparation of NCD message for street theatre	Manoj, Prashanth										■	■										
		Orienting Theatre group on NCD message	Prashanth, Bhanu, Praveen,Muna												■									
		Conducting a Street theatre	MO, Praveen,Bhanu,Muna													■	■	■	■	■	■	■	■	■
	Enough Booklets- 500 Nos	Preparation of ATM booklet-Content & Designing	Manoj, Prashanth						■	■														
		Production of ATM booklet- Printing and Procurement	Manoj, Bhanu, Praveen								■													
		Obtain feedbacks from health worker on utility of the materials	Praveen,Muna, Bhanu									■	■											
		Disseminate materials among health workers, ARS and VHSC members	Praveen,Muna, Bhanu									■	■	■	■									
Training Workshop for PHC Health Workers (ANM, AWW, ASHA)	2 No. (1 Actual + 1 Refresher) per PHC per Year for 26 PHCs	Gathering prior expectations of health workers on training	Prashanth,Praveen,Muna, Bhanu	■							■													
		Preparation of training schedule- High focus on formation of NCD patient groups	Manoj, Prashanth								■													
		Identifying resource material & Preparation of training material	Manoj,Maya								■													
		Translation of training material into Kannada	Praveen,Muna, Bhanu									■												
		Planning of logistics and budget for the training session	Praveen,Muna, Bhanu									■												
		Identifying resource persons	Manoj, Prashanth									■												
		Training and post training feedback	Praveen,Muna, Bhanu										■	■	■	■								
		Follow up of Health Worker Workshops	Praveen,Muna, Bhanu														■	■						
Technical Support to PHC MO for Community platform strenthening		Orientation of PHC MO on package of activities-At 26 PHCs	Praveen,Muna, Bhanu										■	■	■	■	■							
		Supervision and Support to ANM/ASHA for creation of patient group	Praveen,Muna, Bhanu														■	■	■					
		Identify each NCD health day theme at PHC	Praveen,Muna, Bhanu,Respective MO														■	■						
		Schedule and conduct a NCD health day at PHC	MOs, ANM															■	■	■				
		Propose an ARS meeting for purchase and prioritisation of NCD medicines at PHC	Praveen,Muna, Bhanu																■	■	■			
ARS Meeting	2-3 per Year	Orient and capacity building of their functions and use of untied funds	PHC MO, ANMs																		■	■	■	■
		Facilitate purchase and prioritsation of NCD medicines for respective PHC	PHC MOs, ANMs																		■	■	■	■
VHSC meeting	2-3 meetings/ VHSC/Year<br>Total VHSC -NCD meetings/PHC/Year	Coordinate with VHSC members to conduct NCD related seesions in those meetings	ASHA																		■	■	■	■
		Spread awareness on NCD prevention and Drugs availability	ASHA																				■	■
Formation of NCD Patient Group- For 26 PHCs	At least 10 NCD groups per Sub centre	Support ASHA/ANMs to identify and organise NCD patients into a group	Praveen,Bhanu, Munna, MO														■	■	■	■				
		Facilitate Patient groups to attend NCD group meeting, ARS meeting and VHSC meeting	ANM, ASHA																		■	■	■	■
	2-3 NCD group meetings /SC/Year	Conducting NCD patient group Meeting	ANM																		■	■	■	■
Advocacy and Coordination	As and When Required	Meeting with DC, CEO & DHO - Permission letters	Manoj, Prashanth, Team							■														
	Four Times a Year	Meeting district health team-DHO & team -Discuss plan and progress quarterly once	Praveen, Muna, Bhanu		■													■						
	Four Times a Year	Meeting state health team- ED KHSRC, SSO, State Directorate	Manoj, Prashanth			■													■					
	Twice in a Year	Meeting with District warehouse team	Praveen, Muna, Bhanu																					
	Twice in a Year	Meeting with State drug & logistics officer, State pharmacy Council	Manoj																					
	Monthly	Meeting District NCD Officer	Praveen, Muna, Bhanu			■				■					■					■				■
	Four Times a Year	Meeting State NCD Officer	Manoj																					
Training of MOs	One Training course/ PHC/ Year	Gathering prior expectations of MOs on training	Praveen, Muna, Bhanu		■	■																		
		Preparation of training schedule- High focus on adherance to STG, Rational Prescription	Manoj, Prashanth															■	■					
		Identifying resource material & Preparation of training material	Manoj, Prashanth															■	■					
		Planning of logistics and budget for the training session	Praveen, Muna, Bhanu																	■				
		Identifying resource persons	Manoj, Prashanth																	■				
		Training and post training feedback	Praveen, Muna, Bhanu																		■	■	■	■
Training of Pharmacists/ Lab Technicians	One Training course/ PHC/ Year	Gathering prior expectations of Pharmacists on training	Praveen, Muna, Bhanu														■							
		Preparation of training schedule- High focus on Indenting of drugs, Record keeping, Quality assurance	Manoj, Prashanth															■	■					
		Identifying resource material & Preparation of training material	Prashanth, Bhanu															■	■					
		Translation of training material into Kannada	Praveen, Bhanu, Munna																	■				
		Planning of logistics and budget for the training session	Praveen, Bhanu, Munna																	■				
		Identifying resource persons	Bhanu, Prashanth																	■				
		Training and post training feedback	Praveen, Bhanu, Munna																					
