# Supplementary material for: Improving access to medicines for non-communicable diseases in rural India: a mixed methods study protocol using quasi-experimental design
Source: BMC Health Serv Res. 2016 Aug 22;16(1):421. doi: 10.1186/s12913-016-1680-3 (PMC4994301; doi:10.1186/s12913-016-1680-3)
Supplement: Additional file 4: — Household survey tool. Household survey tool used in baseline survey of the ATM study. (PDF 159 kb) [file 12913_2016_1680_MOESM4_ESM.pdf]

# Improving equitable access to quality generic medicines for patients with NCD in Tumkur, India

## Household Survey form - ATM Project

| | | | | | |
|---|---|---|---|---|---|
| A 1 | Door No. | ☐☐-☐☐-☐☐☐☐ | | | |
| A 2.1 | Date of Survey (DD/MM/YYYY) | ☐☐/☐☐/13 | A 2.3 | Team Leader's Code TL ☐ | 1–3 |
| A 2.2 | Time of Survey | ☐☐:☐☐ am/pm | A 2.4 | Area Supervisor's Code F ☐☐ | 01–12 |

**The "Household Informant" should be the person in the household who is the main health care decision maker. This is usually the person who is the most knowledgeable about the health, healthcare expenditures, and healthcare utilization of members of the household. The survey should not be completed if this person, or appropriate substitute, is absent.**

*There is at least one member in this household with Diabetes and/or hypertension AND the person who makes decisions about healthcare in this household, or appropriate substitute, is available to answer:*

**1** ☐ Yes → if Yes for both, Continue **2** ☐ No → if No, stop here

## Access to and use of medicines for NCD Part 1: Household roster

1. Please give the name, sex and age, relationship to head of household, education, occupation, and marital status of each of the household members who live here. Let me assure you that any information you provide will be kept confidential. *Write one person per row and use codes provided in each column*

| | A | B | C | D | E | F | G |
|---|---|---|---|---|---|---|---|
| | **Name** | **Sex** | **Age** | **Relationship to head** | **Education** | **Occupation** | **Marital status** |
| R. No. | Name of each member | 1 = Male<br>2 = Female | Write number of years. Round off to the nearest year. <1 year old is taken as 1. | 1=Head 2=Spouse<br>3=Father 4=Mother<br>5=Son 6=Daughter<br>7=Brother 8=Sister<br>9=Father in law<br>10=Mother in law<br>11=Son in law<br>12-Daughter in law<br>13=Brother in law<br>14=Sister in law<br>15=Grand Son<br>16=Grand Daughter<br>17=Grand Father<br>18=Grand Mother<br>19=Others (specify) | 1=No formal schooling<br>2=Some primary<br>3=Completed Primary<br>4=Completed high school or equivalent<br>5=Completed College/PU/University<br>6=Completed PG<br>7=Not applicable | 1=Farmer/Fisherman<br>2=Office worker<br>3=Civil Servant<br>4=Agric. labour<br>5=Non Agri labour<br>6=Health worker<br>7=Self-employed/own business<br>8=Student/pupil<br>9=Not in labour force/retired<br>10=Other (specify)<br>11=Not Applicable | 1=Married<br>2=Consensual union<br>3=Divorced<br>4=Separated<br>5=Widowed<br>6=Never married<br>7=Non applicable |
| **1** | | | | | | | |
| **2** | | | | | | | |
| **3** | | | | | | | |
| **4** | | | | | | | |
| **5** | | | | | | | |
| **6** | | | | | | | |
| **7** | | | | | | | |
| **8** | | | | | | | |
| **9** | | | | | | | |
| **10** | | | | | | | |
| **11** | | | | | | | |
| **12** | | | | | | | |

| | | | |
|---|---|---|---|
| **1.1** Name of Informant | | **1.2** Relation with head of the household | |
| **1.3** Name of the Area | | **1.4** Name of GP | |
| **1.5** Contact No. | | **1.6** Landmark | |

| | |
|---|---|
| **1.7** Distance of household from the PHC | ☐ <5 k.m. |
| | ☐ 5 to 10 k.m. |
| | ☐ >10 k.m. |

## Access to and use of medicines for NCD Part 2: Health services access and Illness

| 2. How much time does it take to reach the following healthcare facilities or providers that are closest to your household? *Read responses and tick one box for each of the categories.* | **<15 min** | **15 min to 1 hr** | **>1 hr** |
|---|---|---|---|
| Government hospital (CHC, Taluk Hospital, District Hospital) | 1 ☐ | 2 ☐ | 3 ☐ |
| NGO hospital (NGO Hospital) | 1 ☐ | 2 ☐ | 3 ☐ |
| PHC | 1 ☐ | 2 ☐ | 3 ☐ |
| Naati vaidya (trad'l healer) | 1 ☐ | 2 ☐ | 3 ☐ |
| Private pharmacy | 1 ☐ | 2 ☐ | 3 ☐ |
| Private hospital, clinic or physician | 1 ☐ | 2 ☐ | 3 ☐ |

3. In your household, please list the names of people who have **ever been** told by a doctor or other healthcare provider that they have Diabetes or Hypertension?

*Transcribe name and roster number from the household roster*

| **Name** | **Roster number** | **Chronic disease module completed** | |
|---|---|---|---|
| | | **Yes** | **No** |
| | | 1 ☐ | 0 ☐ |
| | | 1 ☐ | 0 ☐ |
| | | 1 ☐ | 0 ☐ |
| | | 1 ☐ | 0 ☐ |

*Complete the next module for each person with a chronic disease (Diabetes/Hypertension or both), One person per page. After collecting complete information about one person, check "Yes" under "Chronic disease module completed" in the corresponding row above. When chronic disease modules are completed and checked for all members listed above, continue on to Question 6. The number of completed "Chronic disease module" pages must equal the number of members with chronic diseases.*

**Chronic**

| Name of person with Diabetes and/or Hypertension | Roster number | |
|---|---|---|
| | | |
| 1. Which illness does (name of the person) have? *Tick both if s/he responds "Yes" to both* **Yes No** | **Yes** | **No** |
| A. Diabetes | 1 ☐ | 0 ☐ |
| B. Hypertension | 1 ☐ | 0 ☐ |
| C. Other chronic illness in addition to the above | | |
| 1. Heart Problem | 1 ☐ | 0 ☐ |
| 2. Stroke | 1 ☐ | 0 ☐ |
| 3. Cancer | 1 ☐ | 0 ☐ |
| 4. Respiratory Problem | 1 ☐ | 0 ☐ |

5. Has (name of the person) been told by the doctor or other healthcare provider that s/he should be taking medicines for his/her illness?

**1** ☐ **Yes** **0** ☐ **No**

6. Do you have any medicines available at home today?

**1** ☐ **Yes** **0** ☐ **No → if No, go to question 9**

7. Which medicines has (name of the person) been told to take for this illness? Can I see that?

*Write one medicine per row, and use codes provided in each column to collect information about each medicine*

| | A | B | C | D | E | F | | G | | H | |
|---|---|---|---|---|---|---|---|---|---|---|---|
| | **Medicine** | **Condition for which medicine was recommended** | **Obtained from** | **Number of days of supply usually obtained** | **Cost for last month** | **Any amount of last month cost covered by insurance or any govt. Scheme?** | | **Label OK** | | **Primary Package Ok** | |
| | Write name of medicine. If name is not known, write the most detailed category given by respondent<br>("antidiabetic", BP medicine etc.) | Write code provided in Q4 above. | 1=Family, friend<br>2=Govt. hosp.<br>3=NGO/mission hospital<br>4=PHC<br>5=Private healthcare provider<br>6=Traditional healer<br>7=Private pharmacy<br>99=Other (specify) | In days. Write "0" if medicine is not obtained regularly | Rounded to the nearest rupee. Write "0"<br>if not obtained regularly or obtained free | Tick Yes if insurance/any government scheme covers part or all cost.<br>Tick No if not obtained or cost not covered by insurance/ government scheme | | Tick Yes if label includes medicine name, dose, and expiration date<br>*Otherwise tick No.* | | Tick Yes if primary package is an envelope or a closable container and if it contains only one medicine<br>*Otherwise tick No.* | |
| | | | | | | **Yes** | **No** | **Yes** | **No** | **Yes** | **No** |
| **Med 1** | | | | | | 1 ☐ | 0 ☐ | 1 ☐ | 0 ☐ | 1 ☐ | 0 ☐ |
| **Med 2** | | | | | | 1 ☐ | 0 ☐ | 1 ☐ | 0 ☐ | 1 ☐ | 0 ☐ |
| **Med 3** | | | | | | 1 ☐ | 0 ☐ | 1 ☐ | 0 ☐ | 1 ☐ | 0 ☐ |
| **Med 4** | | | | | | 1 ☐ | 0 ☐ | 1 ☐ | 0 ☐ | 1 ☐ | 0 ☐ |
| **Med 5** | | | | | | 1 ☐ | 0 ☐ | 1 ☐ | 0 ☐ | 1 ☐ | 0 ☐ |
| **Med 6** | | | | | | 1 ☐ | 0 ☐ | 1 ☐ | 0 ☐ | 1 ☐ | 0 ☐ |
| **Med 7** | | | | | | 1 ☐ | 0 ☐ | 1 ☐ | 0 ☐ | 1 ☐ | 0 ☐ |
| **Med 8** | | | | | | 1 ☐ | 0 ☐ | 1 ☐ | 0 ☐ | 1 ☐ | 0 ☐ |
| **Med 9** | | | | | | 1 ☐ | 0 ☐ | 1 ☐ | 0 ☐ | 1 ☐ | 0 ☐ |

**All Medicines** *(Ask for the total cost of medicines only if the cost of each medicine is not known)*

8. Sometimes people cannot take all medicines as directed. Does (name of person) usually take all medicines as recommended?

1 ☐ **Yes → If Yes, this module is now complete, go back to Question 3.** 2 ☐ **No**

9. If answer to Question 8 is No, ask the following question:

I am going to give you some possible reasons why (name of the person) may not always take medicines as recommended. Can you tell me whether these are the reasons why s/he does not take medicines?

*Read statements, and tick one box for each statement*

| | | **Yes** | **No** |
|---|---|---|---|
| a. | Symptoms have gotten better | 1 ☐ | 0 ☐ |
| b. | Someone in the household decided medicines were not needed | 1 ☐ | 0 ☐ |
| c. | Someone advised not to take medicines | 1 ☐ | 0 ☐ |
| d. | S/he had bad reactions to medicines in the past | 1 ☐ | 0 ☐ |
| e. | Someone in the household chose a different treatment | 1 ☐ | 0 ☐ |
| f. | The place where medicines can be obtained is too far away | 1 ☐ | 0 ☐ |
| g. | Medicines are not available at the PHC/government hospital | 1 ☐ | 0 ☐ |
| h. | Medicines are not available in the private pharmacy or shops | 1 ☐ | 0 ☐ |
| i. | Nobody in the household can take time to obtain medicines | 1 ☐ | 0 ☐ |
| j. | Our household cannot afford the medicines | 1 ☐ | 0 ☐ |
| k. | Other (please specify): | 1 ☐ | 0 ☐ |

**The CHRONIC module is now complete. Go back to Question 3.**

## Access to and use of medicines for NCD
## Part 4: Opinions about obtaining medicines

10. I am going to read you a series of questions about price and quality of medicines. For each question, please tell me YES or No.

*Do not read the option "Do not know". Tick it if the respondent does not want to answer or is unable to choose between "YES" and "NO". Read questions and tick one box for each statement.*

| | | Yes | No | Do not know |
|---|---|---|---|---|
| a. | In government facilities, do the health providers will take into account of your ability to pay when they decide which medicines to be prescribe? | 1 ☐ | 2 ☐ | 3 ☐ |
| b. | In private pharmacy/shop, do they take into account of your ability to pay when they decide which medicines to be prescribe? | 1 ☐ | 2 ☐ | 3 ☐ |
| c. | When you receive a prescription, are you comfortable to ask how much the medicines will cost? | 1 ☐ | 2 ☐ | 3 ☐ |
| d. | Is it easy to you to find out how much medicines cost? | 1 ☐ | 2 ☐ | 3 ☐ |
| e. | Are two identical medicines may be sold at different prices? | 1 ☐ | 2 ☐ | 3 ☐ |
| f. | Do you know where to find medicines at the lowest price nearby? | 1 ☐ | 2 ☐ | 3 ☐ |
| g. | When you buy a medicine, did you ask for the least expensive product? | 1 ☐ | 2 ☐ | 3 ☐ |
| h. | When a pharmacist recommends a medicine, will you be sure that it is the best value for money? | 1 ☐ | 2 ☐ | 3 ☐ |
| i. | When a pharmacist recommends a medicine, will you be sure that it is of good quality? | 1 ☐ | 2 ☐ | 3 ☐ |
| j. | Do you think medicines of better quality are more expensive? | 1 ☐ | 2 ☐ | 3 ☐ |
| k. | Are there any places nearby where you would never buy medicines because they sell medicines of poor quality? | 1 ☐ | 2 ☐ | 3 ☐ |
| l. | Did your government makes sure that the medicines at the PHC/government hospital are of good quality? | 1 ☐ | 2 ☐ | 3 ☐ |
| m. | Do you think different names may be used for the same medicine? | 1 ☐ | 2 ☐ | 3 ☐ |
| n. | Have you heard the word "generic" before to describe a medicine?<br><br>**If respondent has not heard about generic or does not know, skip to question 11.** | 1 ☐ | 2 ☐ | 3 ☐ |
| o. | Is a generic medicine is usually of lower quality than a brand medicine? | 1 ☐ | 2 ☐ | 3 ☐ |
| p. | Is a generic medicine is usually lower in price than a branded medicine? | 1 ☐ | 2 ☐ | 3 ☐ |

## Access to and use of medicines for NCD Part 5: Experiences about medicines

I am going to read you a series of questions about three topics related to care and medicines: access, affordability, and quality. There are no correct answers. For each question, please tell me YES or NO.

*Do not read the option "Do not know". Tick it if the respondent does not want to answer or is unable to choose between "YES" and "NO". Read statements, and tick one box for each statement.*

| **The first set of opinions are about access to care and medicines.** | | **Yes** | **No** | **Do not know** |
|---|---|---|---|---|
| a. | Does the PHC is closest to your house and easy to reach? | 1 ☐ | 2 ☐ | 3 ☐ |
| b. | Does your household members would use PHC pharmacy more if opening hours were convenient? | 1 ☐ | 2 ☐ | 3 ☐ |
| c. | Does the PHC closest to your house usually has the medicines you need? | 1 ☐ | 2 ☐ | 3 ☐ |
| d. | Does the private pharmacy closest to your house usually has the medicines you need? | 1 ☐ | 2 ☐ | 3 ☐ |
| **The second set of opinions are about affordability of medicines** | | **Yes** | **No** | **Do not know** |
| a. | Does your household members can get free medicines at the PHC? | 1 ☐ | 2 ☐ | 3 ☐ |
| b. | Do you think medicines are more expensive at private pharmacies than at PHC? | 1 ☐ | 2 ☐ | 3 ☐ |
| c. | Does your household members can usually afford to buy the medicines you need? | 1 ☐ | 2 ☐ | 3 ☐ |
| d. | Does your household members can usually get credit from the private pharmacy if you need? | 1 ☐ | 2 ☐ | 3 ☐ |
| e. | Does your household members can obtain prescribed medicines from insurance or govt. scheme reimbursed part of the cost? | 1 ☐ | 2 ☐ | 3 ☐ |
| f. | In the past, does your household had to borrow money or sell things to pay for medicines? | 1 ☐ | 2 ☐ | 3 ☐ |
| **The last set of opinions are about quality of care and medicines** | | **Yes** | **No** | **Do not know** |
| a. | Do you think the quality of services delivered at the PHC nearby is good? | 1 ☐ | 2 ☐ | 3 ☐ |
| b. | Do you think the quality of services delivered by the private doctor/clinic nearby is good? | 1 ☐ | 2 ☐ | 3 ☐ |
| c. | Do you think imported medicines are of better quality than locally manufactured medicines? | 1 ☐ | 2 ☐ | 3 ☐ |

## Access to and use of medicines for NCD Part 6: Assets and medicines expenditures

Finally I would like to ask a few questions about the possessions that are in your home and how much your household spends. Remember that any information you provide will be kept confidential.

| | |
|---|---|
| 14. Can you please tell me how many rooms (bedrooms, dining room, kitchen…) are in your home? | ................Rooms |

15. Does anyone in your household have:
*(These items will be added to reflect social status/economic status)*

| | | Yes | No | | | Yes | No |
|---|---|---|---|---|---|---|---|
| a. | House (own) | 1 ☐ | 0 ☐ | d. | Landline | 1 ☐ | 0 ☐ |
| b. | Land (own) | 1 ☐ | 0 ☐ | e. | 4 Wheeler | 1 ☐ | 0 ☐ |
| c. | 2 Wheeler | 1 ☐ | 0 ☐ | | | | |

16. Does your household have:

| | | | | | | | |
|---|---|---|---|---|---|---|---|
| a. | Tap/running water inside the house | 1 ☐ **Yes** | 0 ☐ **No** | c. | Electricity | 1 ☐ **Yes** | 0 ☐ **No** |
| b. | Toilet | 1 ☐ **Yes** | 0 ☐ **No** | → **If NO electric power supply, skip to question 18** | | | |

17. Does your household have?

| | | | | | | | |
|---|---|---|---|---|---|---|---|
| a. | TV | 1 ☐ **Yes** | 0 ☐ **No** | c. | Fan | 1 ☐ **Yes** | 0 ☐ **No** |
| b. | Radio | 1 ☐ **Yes** | 0 ☐ **No** | d. | Mobile | 1 ☐ **Yes** | 0 ☐ **No** |

| | |
|---|---|
| 18. In the last month, how much did your household spend on food? Include the value of any food produced and consumed by the household; exclude alcohol, tobacco, and restaurant meals | **Rs.** ........................................ |
| 19. Please choose the level that is closest to what your household spent in total over the past month. *(Select the ranges of expenditures as shown below)* | ☐ **A**= Rs.1000 to 3000<br>☐ **B**= Rs. 3001 to 5000<br>☐ **C**= Rs. 5001 to 7000<br>☐ **D**= Rs. 7001 to 10000<br>☐ **E**= Rs. 10001+ |

20. In the last 4 weeks, how much did your household spend on:

| | | |
|---|---|---|
| a. | Admitted overnight in the hospital | |
| b. | Medicines | |
| c. | Any other healthcare products or services those were not included above (outpatient visits, lab tests, x ray, ear or eye care etc.) | |
| d. | Voluntary health insurance or payment for any govt. health scheme | |

| | |
|---|---|
| 21. Who is the main earner in the household? *Write his/her roster number* | |

***Thank the interviewed person and reassure about the confidentiality of his/her answers***

22) House type

22.1) Kaccha House (hut) ☐

22.2) Pakka House (Mold or stone built house) ☐

22.3) SemiPakka House (Sheet house, Tile house) ☐

23) Do you have cattle in your home? Yes ☐ No ☐

23.1) Ox (Specify the Numbers) ☐

23.2) Cow /buffalo ☐

23.3) Sheep/Goat ☐

23.4) Hen ☐

24) Agricultural land details (In acres, specify the numbers)

24.1) Own land (in acres) ☐

24.2) Lease or rented land ☐

25) Cast ____________________________________________________

25.1) Religion/Sub cast___________________

26) Ration card details Yes ☐ No ☐

26.1) BPL ☐

26.2) APL ☐

26.3) Anthyodaya ☐

27) Total income of the family per month in Rs _____________________________
