# Supplementary material for: Improving access to medicines for non-communicable diseases in rural India: a mixed methods study protocol using quasi-experimental design
Source: BMC Health Serv Res. 2016 Aug 22;16(1):421. doi: 10.1186/s12913-016-1680-3 (PMC4994301; doi:10.1186/s12913-016-1680-3)
Supplement: Additional file 5: — Facility survey tool. Facility survey tool used in baseline survey of the ATM study. (PDF 318 kb) [file 12913_2016_1680_MOESM5_ESM.pdf]

IMPROVING EQUITABLE ACCESS TO QUALITY GENERIC MEDICINES FOR PATIENTS WITH NCD IN TUMKUR, INDIA

Institute of Public Health (Bangalore), Karnataka
Health Systems Resource Centre (Bangalore) & Institute of Tropical Medicine, Antwerp, Belgium.
Supported by WHO-Alliance, Geneva

***Survey form 1*: PHC Pharmacy** 1

Indicator: Availability of key medicines % medicines expired

Key medicines to treat Diabetes/Hypertension [A]		In stock Yes=1, No=0 [B]	Expired medicines on shelves Yes=1, No=0 [C]	Remarks
1.	TAB HYDROCHLORTHIAZIDE(50mg)			
2.	TAB ATENOLOL(50mg)			
3.	TAB ENALAPRIL(10mg)			
4.	TAB LOSARTAN(50mg)			
5.	TAB AMLODIPINE(5mg)			
6.	TAB ATORVASTATIN(10mg)			
7.	TAB GLIBENCLAMIDE(5mg)			
8.	TAB METFORMIN(500mg)			
9.	TAB PIOGILTAZONE(1mg)			
10.	TAB GLIMEPIRIDE(1mg)			
11.	INJ INSULIN			
		**[B^1] = Sum of B =**	**[C^1] = Sum of C =**	
		**[B^2] = % in stock = $B^1 \div 11 \times 100$ =**	**[C^2] = % expired = $C^1 \div B^1 \times 100$ =**	

Optional additional medicines		In stock Yes=1, No=0	Expired medicines on shelves Yes=1, No=0
1.			
2.			
3.			

Notes:

[A] A list of upto 13 key medicines will be identified and preprinted on the survey forms. This will be done based on the district's drug procurement plan for this year.

[B] Mark “1” if stock is available in the facility on the day of the visit if any quantity of any dosage form is available. Mark “0” if the medicine is not physically available. Add the total at the bottom [B^1]. Calculate the percentage in stock [B^2] by dividing the total in stock [B^1] by 15 and multiplying by 100.

[C] For all medicines in stock, check if expired or not. If any of the medicine has an expiry problem, mark “1” for yes. Do not count expired medicines stored in a separate area for destruction. Add the total at the bottom [C^1]. Calculate the percentage expired [C^2] by dividing the total expired [C^1] by the total number of medicines in stock [B^1] and multiplying by 100.

Survey form 2: **PHC Pharmacy**	2

Indicator: procurement of Price of key medicines

	**Key medicines to treat Diabetes and Hypertension**<br>[A]	**Unit cost**<br>[B]	**Remarks**<br>[C]
1.	TAB HYDROCHLORTHIAZIDE(50mg)		
2.	TAB ATENOLOL(50mg)		
3.	TAB ENALAPRIL(10mg)		
4.	TAB LOSARTAN(50mg)		
5.	TAB AMLODIPINE(5mg)		
6.	TAB ATORVASTATIN(10mg)		
7.	TAB GLIBENCLAMIDE(5mg)		
8.	TAB METFORMIN(500mg)		
9.	TAB PIOGILTAZONE(1mg)		
10.	TAB GLIMEPIRIDE(1mg)		
11.	INJ INSULIN		

	**Optional additional medicines**	**Preparation and unit**	**Lowest price paid by facility**
1.			
2.			
3.			

Notes:

[A] The list from Survey 1 will be preprinted on this form.

[B] The cost specified as per the rate contract/procurement guideline of the Karnataka state government will be pre-printed here.

[C] For each available medicine, the unit price paid per tablet shall be recorded based on the source of its procurement. PHCs obtain medicines either from the central (federal government) supplies, state / district warehouse, local purchase, ZP fund.

[D] Mention source of drug for ex: Drug with ZP Centre, etc.,

Indicator: Average stock out duration and adequate record keeping

Key medicines to treat Diabetes/Hypertension [A]		Records cover at least 6 months within the past 12 months Yes=1, No=0 [B]	Only collect data for medicines with records covering at least 6 months within the past 12 months			
			Number of days out of stock [C]	Number of days covered by the review (at least 6 months) [D]	Equivalent Remark number of days per year [E]=Cx365 ÷ D [E]	Remarks
1.	TAB HYDROCHLORTHIAZIDE(50mg)					
2.	TAB ATENOLOL(50mg)					
3.	TAB ENALAPRIL(10mg)					
4.	TAB LOSARTAN(50mg)					
5.	TAB AMLODIPINE(5mg)					
6.	TAB ATORVASTATIN(10mg)					
7.	TAB GLIBENCLAMIDE(5mg)					
8.	TAB METFORMIN(500mg)					
9.	TAB PIOGILTAZONE(1mg)					
10.	TAB GLIMEPIRIDE(1mg)					
11.	INJ INSULIN					
		**[B^1] = Sum of B =**			**[E^1] = Sum of E =**	
		**[B^2] = % adequate records = B^1 ÷**				
**[F] = Average number of stockout days = E1 ÷ B1 =**						

Key medicines to treat Diabetes/Hypertension		Records cover at least 6 months within the past 12 months Yes=1, No=0	Only collect data for medicines with records covering at least 6 months within the past 12 months		
			Number of days out of stock	Number of days covered by the review (at least 6 months)	Equivalent number of days per year [E] = C x 365 ÷ D
1.					
2.					
3.					

Notes:

[A] The list from Survey 1 will be preprinted on this form.

[B] Go through the stock cards and indicate which medicines have records covering at least 6 months within the previous 12 months. Add the total at the bottom [B^1]. Calculate the percentage of medicines with adequate records [B^2] by dividing the number of medicines with records covering at least 6 months [B^1] by 15 and multiplying by 100.

[C] The review should cover 6-12 months. Go through the stock cards covering the review period. Indicate the number of days each medicine was not available or marked "0" on the card. A medicine is considered in stock if any quantity of it is available in generic or branded form.

[D] Indicate the number of days actually reviewed for each medicine.

[E] Compute the equivalent number of stockout days per year for each medicine by multiplying the number of days out of stock [C] by 365 and dividing by the number of days covered by the review [D]. Add the total number of stockout days [E^1].

[F] Calculate the average number of stockout days by dividing the total number of stockout days [E^1] by the total number of medicines reviewed [B^1].

Example:

Key medicines to treat common conditions [A]	Records cover at least 6 months within the past 12 months Yes=1, No=0 [B]	Only collect data for medicines with records covering at least 6 months in the past 12 months		
		Number of days out of stock [C]	Number of days covered by the review [D]	Equivalent number of days per year [E] = C x 365 ÷ D [E]
Hydrochlogothiazide	1	90	180	182.5
Metformin	1	30	365	30
Any form of Injectable Insulin	0			
	[B^1] = Sum of B = 2			[E^1] = Sum of E =212.5
	[B^2] = % adequate records = B^1 ÷ 3 x 100 = 66.7			
[F] = Average number of stockout days = E^1 ÷ B^1 = 106.25				

Indicator: Adequate conservation conditions and handling of medicines

	**Checklist**	**Storeroom True=1, False=0** [A]	**Dispensing Area/Room True=1, False=0** [B]
1.	There is a method in place to control temperature (e.g. roof and ceiling with space between them in hot climates).		
2.	There are windows that can be opened or there are air vents.		
3.	No direct sunlight can enter the area (e.g. windowpanes are painted or there are curtains/blinds to protect against the sun).		
4.	Area is free from moisture (e.g. leaking ceiling, roof, drains, taps, etc.).		
5.	Medicines are not stored directly on the floor.		
6.	In the facility there is a cold storage with temperature chart.*		
7.	Medicines are stored in a systematic way (e.g. alphabetical, pharmacological or first expiry-first out).		
8.	There is no evidence of pests in the area.		
9.	Tablets/capsules are not manipulated by naked hand.		
		**[A^1] = Sum of A =**	**[B^1] = Sum of B =**
		**[A^2] = Score = $A^1 \div 8 \times 100$ =**	**[B^2] = Score = $B^1 \div 8 \times 100$ =**

Notes:

[A] Indicate "1" if all parts of the statement are true for the storeroom and "0" if any part of it is false. Sum the total number of true statements [A^1]. Calculate the score for the storeroom [A^2] by dividing the sum of true statements [A^1] by 8 and multiplying by 100.

[B] Indicate "1" if all parts of the statement are true for the dispensing area/room and "0" if any part of it is false. Sum the total number of true statements [B^1]. Calculate the score for the dispensing area [B^2] by dividing the sum of true statements [B^1] by 8 (number of applicable statements for each storage area recorded on form) and multiplying by 100.

* It may be necessary to look elsewhere in the facility for some of the criteria (e.g. Refrigerator)

Indicators: Availability of Standard Treatment Guidelines (STG)
Availability of Essential Medicines List (EML)

**Standard Treatment Guidelines (STG) available**	**Yes=1, No=0** [A]
STG for Diabetes (as part of combined STG publication or disease specific STG document)	
STG for Hypertension (as part of combined STG publication or disease specific STG document)	
**[A[1]] =Both STGs are present =**	

**Essential Medicine List (EML) updated within last 5 years available**	**Yes=1, No=0** [B]
State EML	
Provincial/District EML	
Facility-specific EML	
**[B[1]] =At least one current EML is present =**	

Notes:

[A] Identify at the national level and preprint on the form the second required STG. This should be for an important disease in the region, e.g. malaria in Africa. Check to see if there is a copy of each of the STGs either as part of combined STG publication or disease specific STG document. Record "1" if the facility is able to present a copy of the document and "0" if the facility is unable to present the document. If both STGs are present record "1" in [A[1]] otherwise record "0".

[B] Record "1" next to each type of EML that is both physically present in the facility and updated within the past five years. If the facility is unable to present the document or it has been more than 5 years since it was last updated, record "0". If any current EML is available, mark "1"in [B[1]], otherwise record "0".
