# Supplementary material for: Improving access to medicines for non-communicable diseases in rural India: a mixed methods study protocol using quasi-experimental design
Source: BMC Health Serv Res. 2016 Aug 22;16(1):421. doi: 10.1186/s12913-016-1680-3 (PMC4994301; doi:10.1186/s12913-016-1680-3)
Supplement: Additional file 6: — Exit interview tool. Exit interview tool used in baseline survey of the ATM study. (PDF 150 kb) [file 12913_2016_1680_MOESM6_ESM.pdf]

Survey form 5: PHC Exit Interview | 5

Indicators: Average number of medicines per NCD prescription % patients know how to take NCD medicines % NCD medicines dispensed or administered.

Patient sex M/F [A]		Number of medicines prescribed [B]	Number of medicines dispensed or administered [C]	Number of medicines adequately labeled [D]	Patient knows how to take medicines(frequency and duration) Yes=1, No=0 [E]	How long did it take to the patient to reach the hospital 1.<30min 2.31min-1hr 3.>1hr [F]	How much did it cost to come (travel etc) [G]
1.							
2.							
3.							
4.							
5.							
6.							
7.							
8.							
9.							
10.							
11.							
12.							
13.							
14.							
15.							
16.							
17.							
18.							
19.							
20.							
21.							
22.							
23.							
24.							
25.							
26.							
27.							
28.							
29.							
**$[A^1]$ = Sum cases =**		**$[B^1]$ = Sum of B =**	**$[C^1]$ = Sum of C =**	**$[D^1]$ = Sum of D =**	**$[E^1]$ = Sum of E =**	**$[F^1]$ = Sum of F =**	**$[G^1]$ = Sum of G =**
**$[A^2]$ = Sum females =**		**$[B^2]$ = Average number of medicines = $B^1 \div A^1$ =**	**$[C^2]$ = % dispensed = $C^1 \div B^1 \times 100$ =**	**$[D^2]$ = % adequately labeled = $D^1 \div C^1 \times 100$ =**	**$[E^2]$ = % know how to take medicines = $E^1 \div A^1 \times 100$ =**	**[H] = Average cost = $(F^1 + G^1) \div A^1$ =**	
**$[A^3]$ = % females = $A^2 \div A^1 \times 100$ =**							

Notes:

[A] Interview up to 30 patients leaving the dispensing area/pharmacy. These 30 patients shall have obtained treatment either for Diabetes/Hypertension. Record the number of cases [A^1] and the number of females [A^2]. Calculate the percentage of females by dividing the total number of females [A^2] by the total number of cases [A^1] and multiplying by 100.

[B] Record the number of medicines (chemical entity, INN, generic) prescribed for each patient. Combination medicines in one dosage form count as one medicine. Sum the number of medicines prescribed for all patients [B^1]. Calculate average number of medicines prescribed [B^2] by dividing number of medicines prescribed [B^1] by number of cases [A^1].

[C] Record the number of medicines dispensed or administered to each patient. Sum the total number [C^1]. Calculate the percentage of medicines dispensed [C^2] by dividing the number of medicines given to all patients [C^1] by the total number of medicines prescribed [B^1] and multiplying by 100.

[D] Record the number of medicines labelled with at least the name of the medicine and how to take it. Count only medicines meeting at least both criteria. Total the number [D^1]. Calculate the percentage of medicines adequately labelled [D^2] by dividing the total number of adequately labelled medicines [D^1] by the total number of medicines dispensed [C^1] and multiplying by 100.

[E] Determine if patient knows how to take all medicines dispensed. Mark “1” only if patient can correctly state how ALL medicines should be taken and “0” otherwise. Sum the total [E^1]. Calculate the percentage of patients who know how to take all medicines [E^2] by dividing the total number who know how to take all medicines [E^1] by the total number interviewed [A^1] and multiplying by 100.

[F] Record the amount each patient paid out-of-pocket for the medicines received at the facility. Check with a receipt if possible. Sum the total amount [F^1].

[G] Record the amount of other non-diagnostic fees paid by the patient, such as visit or injection fees but not lab or x-ray fees. Sum the total amount [G^1]. [H]

Calculate the average amount cost by adding the amounts paid for medicines [F^1] and fees [G^1] and dividing by the total number interviewed [A^1].

Indicators: Average number of medicines per prescription % prescribed medicines on EDL
% patients prescribed medicines/injections % medicines prescribed by generic name

S.No [A]	Patient sex M/F [B]	Names of medicines prescribed [C]	Genric equivalent name(content name) [D]	No of tab prescribed [E]	No of tab prescribed, dispensed [F]	Whether the med is on EML (Y/N) [G]
1.						
2.						
3.						
4.						
5.						
	**[B^1] = Sum cases =**	**[C^1] = Sum of C =**	**[D^1] = Sum of D =**	**[E^1] = Sum of E =**	**[F^1] = Sum of F =**	**[G^1] = Sum of G =**
	**[B^2] = Sum females =**<br>**[B^3] = % females = $B^2 \div B^1 \times 100$ =**	**[C^2] = Average number of medicines = $C^1 \div B^1$ =**	**[D^2] = % receiving antibiotics = $D^1 \div B^1 \times 100$ =**	**[E^2] = % receiving injections = $E^1 \div B^1 \times 100$ =**	**[F^2] = % EML = $F^1 \div C^1 \times 100$ =**	**[G^2] = % INN = $G^1 \div C^1 \times 100$ =**

Notes:

[A] From outpatient treatment records, select 30 patients seen within the last 12 months (R = retrospective sampling). If records are not available, select 30 patients currently being treated (P = prospective sampling). Sample can combine R and P. The process is described on page 2728 of *The Manual*. Mark "R" if patient was selected retrospectively and "P" if patient was selected prospectively.

[B] Record the number of cases [B^1] and the number of females [B^2]. Calculate the percentage of females by dividing the total number of females [B^2] by the total number of cases [B^1] and multiplying by 100.

[C] Record number of medicines (chemical entity, INN, generic) prescribed. Combination medicines in one dosage form count as one medicine. Total the number of medicines prescribed [C^1]. Calculate average number of medicines prescribed [C^2] by dividing number of medicines prescribed [C^1] by number of cases [B^1].

[D] Record "1" if patient was prescribed any antibiotics and "0" otherwise. Total the cases receiving antibiotics [D^1]. Calculate percentage of cases with antibiotics [D^2] by dividing number of cases with antibiotics [D^1] by number of cases [B^1] and multiplying by 100.

[E] Record "1" if patient was prescribed any injections and "0" otherwise. Total the cases receiving injections [E^1]. Calculate percentage of cases receiving injections [E^2] by dividing number of cases with injections [E^1] by number of cases [B^1] and multiplying by 100.

[F] Record number of prescribed medicines on the national Essential Medicines List (EML). Total the number of prescribed medicines on the EML [F^1]. Calculate the percentage of prescribed medicines on the EML [F^2] by dividing the number of medicines on the EML [F^1] by the number of medicines prescribed [C^1] and multiplying by 100.

[G] Record number of medicines prescribed by INN. Total the number of medicines prescribed by INN [G^1]. Calculate percentage of medicines prescribed by INN [G^2] by dividing number of medicines prescribed by INN [G^1] by number of medicines prescribed [C^1] and multiplying by 100.
