# Supplementary material for: Improving access to medicines for non-communicable diseases in rural India: a mixed methods study protocol using quasi-experimental design
Source: BMC Health Serv Res. 2016 Aug 22;16(1):421. doi: 10.1186/s12913-016-1680-3 (PMC4994301; doi:10.1186/s12913-016-1680-3)
Supplement: Additional file 7: — Information consent. Information sheet used for obtaining consent in household survey of the ATM study. (PDF 428 kb) [file 12913_2016_1680_MOESM7_ESM.pdf]

Improving access to quality generic medicines for patients with NCD in Tumkur, India

Information sheet for household survey participants

Thank you for considering taking part in this research study. Before you make a decision, it is important for you to understand why this research is being done and what it will involve. Please take time to read the following information carefully and please do not hesitate to ask any team member if there is anything that is not clear, or if you would like more information. Please take time to decide whether or not you wish to participate. Thank you for reading this information.

What is this study?

Institute of Public Health, Bangalore (IPH), Karnataka State Health Systems Resource Centre, Bangalore (KSHSRC) and Institute of Tropical Medicine, Antwerp (ITM) are conducting a research study titled "Improving equitable access to quality generic medicines for patients with Non Communicable Diseases" in Tumkur district, Karnataka. The duration of the project is three years (2013-2016). This research is supported by the WHO-Alliance and will be implemented in three talukas of Tumkur district - Koratagere, Turuvekere and Sira.

What is the Purpose of the Study?

In India, in spite of several efforts at improving access to medicines for diabetes and hypertension, there are still physical and financial barriers to achieving complete access to drugs. This study is being conducted in Tumkur district to understand how this can be improved through improving existing arrangements for community participation in PHCs as well as improving health services at PHCs for providing care for non-communicable diseases.

In this study, we are conducting surveys in the community, interviews with PHC staff, government officials and private pharmacists and test drugs at these places to understand the present level of access to medicines for non-communicable diseases.

Why have you been chosen?

As part of the study, we are conducting a household survey in all three study Talukas. This survey will focus on the health seeking behavior of NCD patients, their health expenditure, and issues around access to medicines including availability and utilization NCD medicines. You have been selected as a respondent for this survey and we would like to ask you certain questions regarding obtaining medicines for hypertension/diabetes, including the difficulties you face. Your knowledge about various issues around access to medicines will help us in planning the interventions to improve access to medicines.

What does participation involve?

If you agree to take part, please sign and give back the consent form to one of the team member. Then you will be part the survey we are conducting in Tumkur district. I would like to confirm that everything you say would be kept private and confidential. Only the researcher will have access to the information you share. Your name will not be used in any research documentation or any discussions related to the research study.

What are the possible benefits of taking part?

There is no direct benefit to you from participation in this research. However, the team believes that the results of the study will contribute to the improvement with regard to awareness, utilisation and access to medicines for non-communicable diseases in Tumkur.

Contact for further information

Dr. N.S Prashanth, Principal investigator

No:250, 2nd C Main,

2nd C Cross, Girinagar I Phase, Bangalore 560085

Email: prashanthns@iphindia.org. Phone: 91 80 2642 1929

Alliance for Health Policy and Systems Research

NATIONAL RURAL HEALTH MISSION
GOVERNMENT OF KARNATAKA
KSHSRC

Institute of Public Health, Bengaluru

Improving equitable access to quality generic medicines for patients with NCD in Tumkur, India

Consent form for Participation in Household Survey

Please tick box

1. I confirm that I have read and understood the information sheet and have had the any related questions answered satisfactorily.

   ☐ YES ☐ NO

2. I understood that my participation is voluntary and that I am free to withdraw at anytime without giving any reason.

   ☐ YES ☐ NO

3. I agree to take part in the above study.

   ☐ YES ☐ NO

Participant's Details

Name --

Address--

Landline Phone /
Mobile Number --

Signature--- Date ---------------------

Thumb impression

Data Collector's Name--

Signature-- Date ---------------------
