# Supplementary material for: Improving access to medicines for non-communicable diseases in rural India: a mixed methods study protocol using quasi-experimental design
Source: BMC Health Serv Res. 2016 Aug 22;16(1):421. doi: 10.1186/s12913-016-1680-3 (PMC4994301; doi:10.1186/s12913-016-1680-3)
Supplement: Additional file 8: — Supervisor structured checklist for household survey. Checklist for the supervisor to validate on field data collection in the household survey of the ATM study. (PDF 75 kb) [file 12913_2016_1680_MOESM8_ESM.pdf]

Validation checklist – ATM household survey

General instructions:

1. Everyday observe 2 field investigators administering questionnaires - one in the morning and another in the afternoon. Note down any mistakes you observe in the way they ask the questions. Next, spend some time clarifying these mistakes to them and help them collect the right information.
2. Select 2 forms from different field investigators daily for validation. If you have time and especially in the first few days of survey, you can always cross-check more forms. Once you select a form, ask the concerned field investigator for directions to the house and return to the house with the filled form.
3. When you go back to the household, explain that you are the supervisor for the field investigators and so you need to cross-check their work. Hence, you will again need to ask them a few questions and take their permission for the same. If they refuse or are not available, pick another form to validate.
4. If the information you collect for any question does not match the information filled in the form, note down the correct information. Next discuss your findings with the concerned field investigator and make sure they enter the correct information after correcting with a whitener.
5. Most importantly remember that the purpose of cross-checking is not to catch mistakes and fault with the field investigator. The purpose is to ensure that the data collected is correct and any mistakes identified will allow you to sit with the concerned field investigator and re-explain the question and how to fill it.

Date:

Name of PHC:

Cluster:

Village:

UID:

**Qn No.**	**Question asked**	**Things to look for- the response**	**Information collected matches with information entered**<br>☑ Yes ☒ No
	Has the ATM project information sheet been given to read or has been read out to the family?	If the response is (No) for any of these questions, then re-explain the project and consent details.	☐
	Was the consent taken in either written or oral format?		☐
	Has the informant understood the purpose of the study?		☐
**A1**	Household number	Should be left blank	☐
**1.**	No. of members in household	Verify how many members are actually permanent members of the household. In case there is any mismatch, then correct the information accordingly	☐
**1.1**	Contact number	Should be entered if available	☐
**5.**	*Is the patient regularly taking medicines?*	If not, make sure they fill question no.8	☐
**7(A)**	Names of medicines	Verify that the names of medicines are entered correctly	☐
**7.1**	Total cost of medicines in last month	Make sure if the respondent does not know it is entered as don't know , not a random number	☐
**13.a**	*Is the quality of services in the nearby PHC good?*	Just validate the response	☐
**20.b**	Last month expense on medicine for the whole family	Make sure it is for whole family and not just about the NCD patient	☐
