# Supplementary material for: Improving access to medicines for non-communicable diseases in rural India: a mixed methods study protocol using quasi-experimental design
Source: BMC Health Serv Res. 2016 Aug 22;16(1):421. doi: 10.1186/s12913-016-1680-3 (PMC4994301; doi:10.1186/s12913-016-1680-3)
Supplement: Additional file 9: — Drug quality tests. This document provides rationale and step by step narration for drug quality testing activities conducted under ATM study. (PDF 54 kb) [file 12913_2016_1680_MOESM9_ESM.pdf]

Drug quality tests

A quality test for NCD medicines has been planned as an independent activity of the study.

Purpose of this activity

One of the common perceptions in the community and even unfortunately within the staffs of the health services in India is that generic medicines are of less quality and having less effect in treating any given disease including NCDs compared to branded medicines available in the market. This has indeed lead to various repercussions on the patients in the community and the health system itself. This has been most often lead to a reduced demand for such drugs from the patients leading to a irregular supply chain management in procuring such drugs by government t PHCs and other facilities. Hence availability of such drugs is a real problem in local health facilities. Patients often buy branded medicines from the private pharmacies from their pocket. This lead to the poor families becomes poorer particularly if a member suffering from chronic disease like diabetes or hypertension and need to be on lifelong medication.

With this concern, this study envisages to conduct quality tests of some of the generic and branded medicines for NCD together. We will see to identify and assess the actual content, weight and other standardized criterions as mentioned on these medicines and see all these met the respective quantity and quality parameters.

1.1.1.1. Sampling points for the medicines

One type of sample medicines will be collected from randomly selected PHCs, and the pharmacy in the government district hospital in three talukas for quality testing. The nearest private pharmacy of the selected PHC will be the sampling point for the private sector. The most widely dispensed proprietary medicine will be the second type of sample and the least expensive unbranded generics will be the third type of sample to be sampled from the private pharmacy. Then a fourth and fifth type of sample will be collected which consists of generics and branded medicines that are only sold in a district neighboring the study district for the whole of the state.

1.1.1.2. Sample size

In all, two medicines each for diabetes and hypertension will be sampled. Five samples for each such medicine will be collected for the quality testing as described above. So totally 20 samples will be collected. The required quantity of samples will be determined.

1.1.1.3. Drug testing laboratories

Sample medicines will be sent to a Government run drug testing lab in the state where the study is happening and to a private drug testing lab in the neighboring state in equal quantity. Both the labs will be prior checked for their accreditation with the standard authority for regulating labs in the country.

1.1.1.4. Standards of reference

All the tests that will be carried out by both the government and private labs will be as per the rules and regulations set by Indian Pharmacopoeia 2014.

1.1.1.5. Permissions and collaboration

Before sending the sample medicines for their quality testing necessary permissions will be obtained from the concerned state authority to do testing in the government lab. Similarly a formal agreement will be made with the private

lab to enter into a non-disclosure collaboration with the institution belonging to the research team regarding testing of medicines and communication of the test results.

1.1.1.6. **Blinding of the medicines**

All the sample medicines will be blinded (single blinding) to those assessing the outcomes (this case the lab personnel) with regards to their manufacturer, source of sampling to avoid any influence on the test results. Only the manufacturing date, expiry date and the quantity of each type of sample will be shared with those assessing the outcome, as these are essential prerequisite to any drug testing activities.

1.1.1.7. Frequency and time of drug testing

This will be done before the intervention and repeated during the end line survey (18 months gap). All the reports of these tests will be shared with the state and district drug controller for their information and action. Wherever counterfeit or defective drugs are found, the particular PHC, private pharmacy as well as the district and taluka health officer shall be immediately informed. The number of such incidents shall be recorded.

1.1.1.8. Drug quality test reports

From drug quality test reports the quality of generic and branded medicines for NCD will be compared through checking variety of indicators like weight and content of the medicine, dissolution, UV assay etc. Similarly results will be compared among that come from a private laboratory and government laboratory. Finally these results will again be matched against the results from the repeat drug testing done towards the end term of the study to understand if drugs quality may have improved in response to governance shifts in course of the project.
